# In Situ Growth of Interfacially Nanoengineered 2D–2D WS_2_/Ti_3_C_2_T_*x*_ MXene for the Enhanced Performance of Hydrogen Evolution Reactions

**DOI:** 10.1021/acsami.3c11642

**Published:** 2024-03-12

**Authors:** Faisal Rasool, Bilal Masood Pirzada, Shamraiz Hussain Talib, Tamador Alkhidir, Dalaver H. Anjum, Sharmarke Mohamed, Ahsanulhaq Qurashi

**Affiliations:** † Department of Chemistry, Khalifa University of Science and Technology, Abu Dhabi 127788, United Arab Emirates; ‡ Center for Catalysis and Separations, Khalifa University of Science and Technology, Abu Dhabi, P.O. Box 127788, United Arab Emirates; § Department of Physics, Khalifa University of Science and Technology, Abu Dhabi 127788, United Arab Emirates

**Keywords:** WS_2_/Ti_3_C_2_T_*x*_ MXene, 2D–2D heterostructure, in situ HF, interfacial engineering, hydrogen evolution reaction, DFT

## Abstract

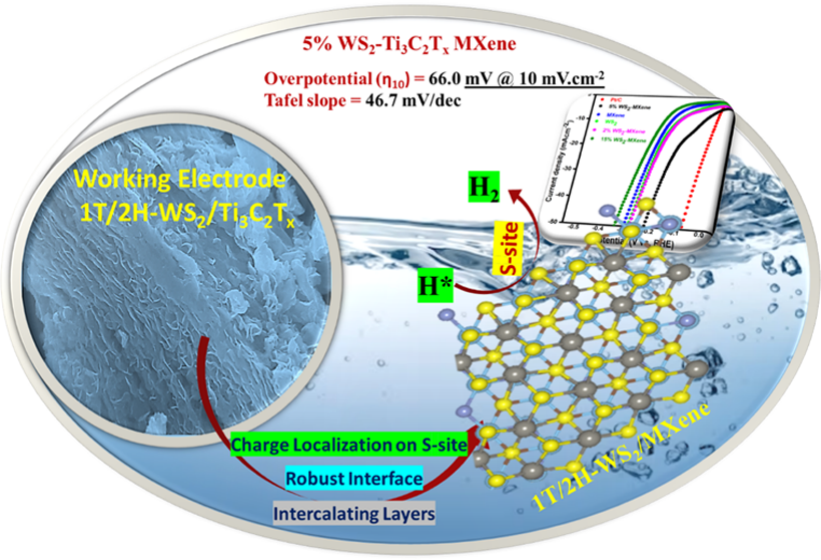

In line with current research goals involving water splitting for hydrogen production, this work aims to develop a noble-metal-free electrocatalyst for a superior hydrogen evolution reaction (HER). A single-step interfacial activation of Ti_3_C_2_T_*x*_ MXene layers was employed by uniformly growing embedded WS_2_ two-dimensional (2D) nanopetal-like sheets through a facile solvothermal method. We exploited the interactions between WS_2_ nanopetals and Ti_3_C_2_T_*x*_ nanolayers to enhance HER performance. A much safer method was adopted to synthesize the base material, Ti_3_C_2_T_*x*_ MXene, by etching its MAX phase through mild in situ HF formation. Consequently, WS_2_ nanopetals were grown between the MXene layers and on edges in a one-step solvothermal method, resulting in a 2D–2D nanocomposite with enhanced interactions between WS_2_ and Ti_3_C_2_T_*x*_ MXene. The resulting 2D–2D nanocomposite was thoroughly characterized using X-ray diffraction (XRD), scanning electron microscopy (SEM), transmission electron microscopy (TEM), Raman, Fourier transform infrared (FTIR), and X-ray photoelectron spectroscopy (XPS) analyses before being utilized as working electrodes for HER application. Among various loadings of WS_2_ into MXene, the 5% WS_2_–Ti_3_C_2_T_*x*_ MXene sample exhibited the best activity toward HER, with a low overpotential value of 66.0 mV at a current density of −10 mA cm^–2^ in a 1 M KOH electrolyte and a remarkable Tafel slope of 46.7 mV·dec^–1^. The intercalation of 2D WS_2_ nanopetals enhances active sites for hydrogen adsorption, promotes charge transfer, and helps attain an electrochemical stability of 50 h, boosting HER reduction potential. Furthermore, theoretical calculations confirmed that 2D–2D interactions between 1T/2H-WS_2_ and Ti_3_C_2_T_*x*_ MXene realign the active centers for HER, thereby reducing the overpotential barrier.

## 1 Introduction

Hydrogen (H_2_) is considered an efficient alternative energy source that can help reduce our reliance on conventional fossil fuels.^1^ It has several advantageous features, including higher gravimetric energy density, zero carbon emissions, eco-friendly, and can be obtained from abundant resources such as water, which make it an increasingly popular choice for energy harvesting.^2^ Also, using H_2_ over the long term could provide an indirect solution to current environmental issues.^3^ Recently, producing green H_2_ through electrocatalytic water splitting has gained huge importance with respect to conventional methods such as coal gasification and natural gas reforming. Despite continuous progress in the electrocatalytic hydrogen evolution reaction (HER), water electrolysis is still facing significant challenges in terms of the efficiency and stability of the synthesized catalytic materials. The most efficient electrocatalyst for HER is platinum (Pt), but its use is limited owing to its higher cost, long-term instability, and limited reserves.^4^ As a result, significant effort has been devoted to exploring earth-abundant electrocatalytic materials that are close or more efficient in performance than Pt in the context of superior activity, selectivity, and stability.^5^

Layered transition metal dichalcogenide (TMD) nanomaterials hold great potential for electrocatalysis owing to enhanced surface-active sites, higher charge transfer, and ease of heterostructure formation with other two-dimensional (2D) or one-dimensional (1D) nanomaterials. Moreover, they exhibit lower toxicity and cost-effectiveness and are earth-abundant.^6,7^ These layered TMD nanomaterials (such as MoS_2_, WS_2_, NbS_2_, etc.) possess out-of-plane sulfur atoms, which easily interact with the surrounding chemical moieties to determine the growth and morphology of heterostructure nanocomposites. Further, the sulfur vacancies present in these TMDs increase the active sites for reactant adsorption, which facilitates the electrocatalytic activity.^8^ To achieve enhanced electrocatalytic performance, a highly conducting support material is needed to facilitate the charge transfer to lower the overpotential for the electrochemical reaction.^9^ MXene, a 2D multilayered material, has excellent conductivity (∼6500 S cm^–1^) and can serve as a good conducting support material for the uniform growth of 2D dichalcogenide sheets over and in-between the layers to obtain a sandwiched wafer-like continuum for enhanced charge transfer and active sites.^10^ However, the inactive basal planes in 2D TMDs sometimes limit their performance.^11,12^ Construction of a 2D–2D heterostructure and interfacial engineering can enhance the charge mobility and reductive behavior at the active sites, which provides faster kinetics for electrocatalysis with higher efficiency and durability.^13,14^

2D–2D heterostructure formation can cause the formation of dissimilar atomic layers with strong covalent bonds that will energize the inert basal plane in single 2D layers and enhance sufficient in-plane stability.^15^ These heterostructures will also offer a variety of active sites and promote efficient transfer of electrons across their interfaces.^16,17^ Many researchers have reported MXene-based heterostructures, such as a ternary hybrid structure of MoS_2_/MXene/CNT, synthesized by Wei et al. using a solvothermal approach.^18^ Similarly, Li et al. employed a hydrothermal strategy for the synthesis of fluorine-free Ti_3_C_2_T_*x*_/MoS_2_, which exhibited an overpotential of 139 mV at −10 mA cm^–2^ with a corresponding Tafel slope of 78 mV dec^–1^ for HER.^19^ Thirumal et al. adopted a hydrothermal approach to synthesize a MXene/reduced graphene oxide (rGO) heterostructure but the results for HER were not very promising.^20^ Previous work by Han et al. proposed the idea of using chemical vapor deposition (CVD) to deposit single vanadium atom layers onto 1T-WS_2_ monolayers, but the results were not close to those for Pt/C, despite being an expensive synthetic method.^5^

Herein, we report for the first time the growth of 1T/2H-WS_2_ nanopetals in-between and over the Ti_3_C_2_T_*x*_ (T_*x*_ = F, Cl or OH) MXene layers, leading to the formation of a 2D–2D nanocomposite using a facile one-step solvothermal method. By mitigating the harshness of acidic HF through in situ generation and controlled etching, we obtained pristine Ti_3_C_2_T_*x*_ MXene. Moreover, microscopic studies revealed the successful integration of 1T/2H-WS_2_ nanosheets (NSs) within the MXene matrix, forming a robust 2D–2D interface. Notably, the proposed 2D–2D interface demonstrated a low overpotential of −66 mV at a current density of −10 mA cm^–2^ in an alkaline electrolyte and a smaller Tafel slope of 46.7 mV·dec^–1^ in the 5% WS_2_–Ti_3_C_2_T_*x*_ MXene sample. Additionally, it exhibited almost a constant overpotential for 50 h of HER at 100 mA cm^–2^, demonstrating its better stability for HER in an alkaline medium.

## 2 Experimental Section

### 2.1 Materials

Tungsten(VI) chloride (WCl_6_; CAS number is 13283–01–7), platinum on carbon (Pt/C; CAS number is 7440–06–4), ammonium fluoride (NH_4_F; CAS number is 12125–01–9), and thioacetamide (H_5_CNS; CAS number is 62–55–5) were purchased from the Sigma-Aldrich Company. A MAX phase precursor for Ti_3_C_2_T_*x*_ MXene was purchased from Jiangsu Xfnano Materials Tech Co., Ltd. China. The reaction solvents such as ethanol (C_2_H_5_OH), hydrochloric acid (HCl), deionized water (H_2_O), acetone (C_3_H_6_O), and *N*,*N*-dimethylformamide (DMF) were acquired from EMSURE Merck.

### 2.2 Methodology

#### 2.2.1 Synthesis of Ti_3_C_2_T_*x*_ MXene by Chemical Etching

Ti_3_C_2_T_*x*_ MXene was acquired through a chemical etching method, which was derived from previously reported methods with minor changes, using the MAX phase (Ti_3_AlC_2_) precursor.^21,22^ Initially, 3 g of Ti_3_AlC_2_ powder was dispersed into 50 mL of a 6 M HCl solution in a high-density poly(tetrafluoroethylene) (PTFE) container. The mixture was stirred using a magnetic stirrer at 50 °C on a hot plate (Thermo Scientific) for half an hour. Subsequently, a certain amount of NH_4_F (a little more than the number of moles of HCl used) was added in small installments to the reaction mixture so as to prevent excessive heat production during the vigorous exothermic reaction of HCl and NH_4_F. The reaction mixture was then vigorously stirred at 50 °C for 24 h to ensure complete etching of the Al layer from the MAX phase. After the reaction was terminated, the mixture was washed multiple times with deionized H_2_O using a benchtop centrifugation device (HERMLE) at 6000 rpm for 10 min in each cycle. The process was repeated many times until the pH of the supernatant was close to ∼7. The as-obtained black sedimented powder was washed with ethanol and then with acetone and was finally dried in an oven at 80 °C to obtain Ti_3_C_2_T_*x*_ MXene powder for further analysis.

Table S1 summarizes the optimization of the molar concentrations of HCl and NH_4_F used in this study for etching the MAX phase. We vary the concentration of HCl while keeping the other parameters constant, such as 50 °C temperature, PTFE container type, deionized H_2_O, a reaction time of 24 h, a centrifugation speed of 6000 rpm, and a stirring rate of 70 rpm.

#### 2.2.2 Synthesis of WS_2_ Nanosheets

For the preparation of WS_2_ NSs, we adopted the synthesis reported previously with slight modifications.^23^ 3 mmol (1.2 g) of WCl_6_ and 30 mmol (2.3 g) of thioacetamide were dissolved in 25 mL of DMF. The reaction mixture was then ultrasonicated for 1 h at 50 °C. Subsequently, the resultant mixture was loaded into a Teflon-lined autoclave (100 mL) and placed inside an oven at 200 °C for 24 h. After completion of the reaction, the precipitate was washed with deionized H_2_O using repetitive centrifugation steps to remove all of the impurities including excessive sulfur. Finally, the sample was washed with ultrapure ethanol and acetone to remove any organic impurities, if any. Subsequently, the WS_2_ precipitate was dried in a vacuum oven at 50 °C for 12 h to obtain the pure WS_2_ powder.

#### 2.2.3 Synthesis of the WS_2_–MXene Nanocomposite

For the preparation of WS_2_–MXene nanocomposite heterostructures, 200 mg of the previously synthesized Ti_3_C_2_T_*x*_ MXene was dispersed in 25 mL of DMF using ultrasonication (DAIHAN Scientific WUC-A03) for 30 min. Then, a solution containing a determined amount of WCl_6_ was added to the mixture and stirred for 30 min, followed by addition of an excessive amount of thioacetamide. Specifically, for the synthesis of the 5% WS_2_–Ti_3_C_2_T_*x*_ MXene nanocomposite, 1.2 g (3 mmol) of WCl_6_ and 2.3 g (30 mmol) of thioacetamide in 25 mL of DMF was added to the MXene dispersion and magnetically stirred for 30 min. The combined reaction mixture was then ultrasonicated for 1 h at 50 °C. Subsequently, the resultant mixture was loaded into a Teflon-lined autoclave (100 mL) and placed inside an oven at 200 °C for 24 h. Once the reaction was complete, the autoclave was allowed to cool to room temperature naturally. The obtained black precipitate was washed several times with deionized H_2_O, then ethanol, and last with acetone. Finally, the as-obtained WS_2_–MXene heterostructure product was dried in an oven at 80 °C for 12 h before being used for further characterization.

Using the same protocol, samples with different WS_2_ loadings of (m/m) 2, 5, and 15% were obtained. All of these synthesized electrocatalysts were analyzed for HER, and the best WS_2_ loading percentage in WS_2_–MXene nanocomposites for HER was established.

### 2.3 Characterization

The series of synthesized electrocatalysts discussed above were characterized using a variety of analytic techniques. The crystalline structure of all of the samples was confirmed using X-ray diffraction (Bruker D2 Phaser XRD with Cu (Kα) radiations). Field emission scanning electron microscopy (JEOL JSM-7610F FE-SEM brand) assembled with energy dispersive X-ray spectroscopy (EDX/EDS) was employed to obtain the morphology, size, and elemental mapping of the synthesized samples and quantification of the constituent elements. The samples were mounted on carbon tape, followed by a gold coating (JEC-3000FC) before being analyzed under an electron microscope. High-resolution transmission electron microscopy (Titan TEM 300 kV), assembled with EDX, was also employed to obtain the morphology, size, and elemental mapping and quantification of the constituent elements. HRTEM was also used to confirm the heterostructure formation, as evident in the alignment of the fringes of different crystalline phases. Further, selected area electron diffraction (SAED) was employed again to confirm heterostructure formation. The samples were further characterized using a Raman microscope (Horiba LabRAM HR Evolution) with an excitation wavelength of 633 nm and a Fourier transform infrared (FTIR) Nicolet IS10 spectrometer (Thermo Scientific) to confirm the presence of various functional group moieties and their interactions in a spectrum range from 750 to 4000 cm^–1^. X-ray photoelectron spectroscopy (XPS, ESCALAB Xi^+^, Thermo Scientific) was also used for the identification of the surface oxidation states with Al–Kα (mono 650 μm).

### 2.4 Electrochemical Measurements

Electrochemical measurements were performed on the prepared electrocatalysts using a Metrohm Multi-Autolab M204 potentiostat. The electrochemical workstation contains a three-electrode cell model where Ag/AgCl (in a 3 M saturated KCl solution) was used as a reference electrode, Pt foil was used as the counter electrode, and the working electrodes were fabricated using the synthesized WS_2_–MXene heterostructure electrocatalysts. The working electrode material was supported on a nickel foam (NF) substrate. The reference electrode potentials were converted using eq (S2) (in the Supporting Information) into the reversible hydrogen electrode (RHE). A single chamber electrolysis cell (100 mL) was used, filled with 25 mL of 1 M KOH as the electrolyte. To deposit the electrode material, a uniform electrocatalyst ink was formed by dispersing 3 mg of the synthesized nanoelectrocatalyst in 500 μL of double-deionized H_2_O with addition of 10 μL of Nafion 117 as the binder. The electrocatalyst mixture was sonicated for 30 min to obtain a uniform ink. The electrocatalyst ink was dropwise cast on the NF substrate (0.5 cm × 0.5 cm) and intermittently left for drying in open air. The fabricated working electrodes were then employed for electrocatalytic water splitting to produce hydrogen gas. The HER activity was evaluated by using linear sweep voltammetry (LSV) at a scan rate of 50 mV s^–1^ with a potential window from −0.8 to −1.5 V versus Ag/AgCl. Tafel slopes were obtained from the LSV results, giving an idea about the HER kinetics. Cyclic voltammetry (CV) was done in the nonfaradaic region, between 0.1 and −0.1 V, at different scan rates from 10 to 100 mV s^–1^, to obtain specific capacitance (*C*_s_) and double-layer capacitance (*C*_dl_) for the determination of electrochemically active surface area (ECSA). Electrochemical impedance spectroscopy (EIS) in the frequency range of 0.01 Hz to 1 × 10^5^ Hz, at an applied voltage of −0.2756 V (vs RHE), was performed on all of the samples. This analysis was conducted to determine the charge transfer resistance (*R*_ct_) from the Nyquist plots. These plots provide insights into the charge transfer characteristics during the electrocatalytic reaction. The electrochemical stability of the applied samples was analyzed using chronoamperometry for HER. All measurements were recorded without any IR compensation or correction. The turnover frequency (TOF) was also calculated using the CV data as shown in the Supporting Information. All of the above electrochemical analyses were done at ambient conditions.

### 2.5 DFT Studies

The density functional theory (DFT) calculations were performed by CASTEP using the plane-wave basis set with ultrasoft pseudopotentials (USP) to describe the ion-electron interaction.^24^ The electron–electron exchange–correlation was accounted for by using the generalized gradient approximation (GGA).^25^ The 400 eV energy cutoff was used for plane-wave expansion, with an optimal force of less than 0.02 eV Å^–1^ and a self-consistent field of 10^–5^ eV. The Brillouin zone grid sampling was tested using the Monkhorst–Pack scheme, and to confirm the desired level of accuracy, 4 × 4 × 1 *k*-points were considered for geometry optimization and total energy calculations. The Tkatchenko–Scheffler correction was employed to describe the van der Waals interactions between the adsorbed hydrogen and structures. A vacuum space of 20 Å was used to prevent the periodic interaction between the periodic images.

The Gibbs free energy of hydrogen adsorption was a key descriptor that was used to evaluate the electrocatalytic HER performance^26^
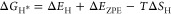
1where Δ*E*_H_ is the adsorption energy of hydrogen and Δ*S*_H_ and Δ*E*_ZPE_ are the differences in entropy and zero-point energy between the atomic hydrogen absorption and the gas phase hydrogen, respectively, which are calculated by the vibration frequencies of the system.

## 3 Results and Discussion

**Scheme 1:**
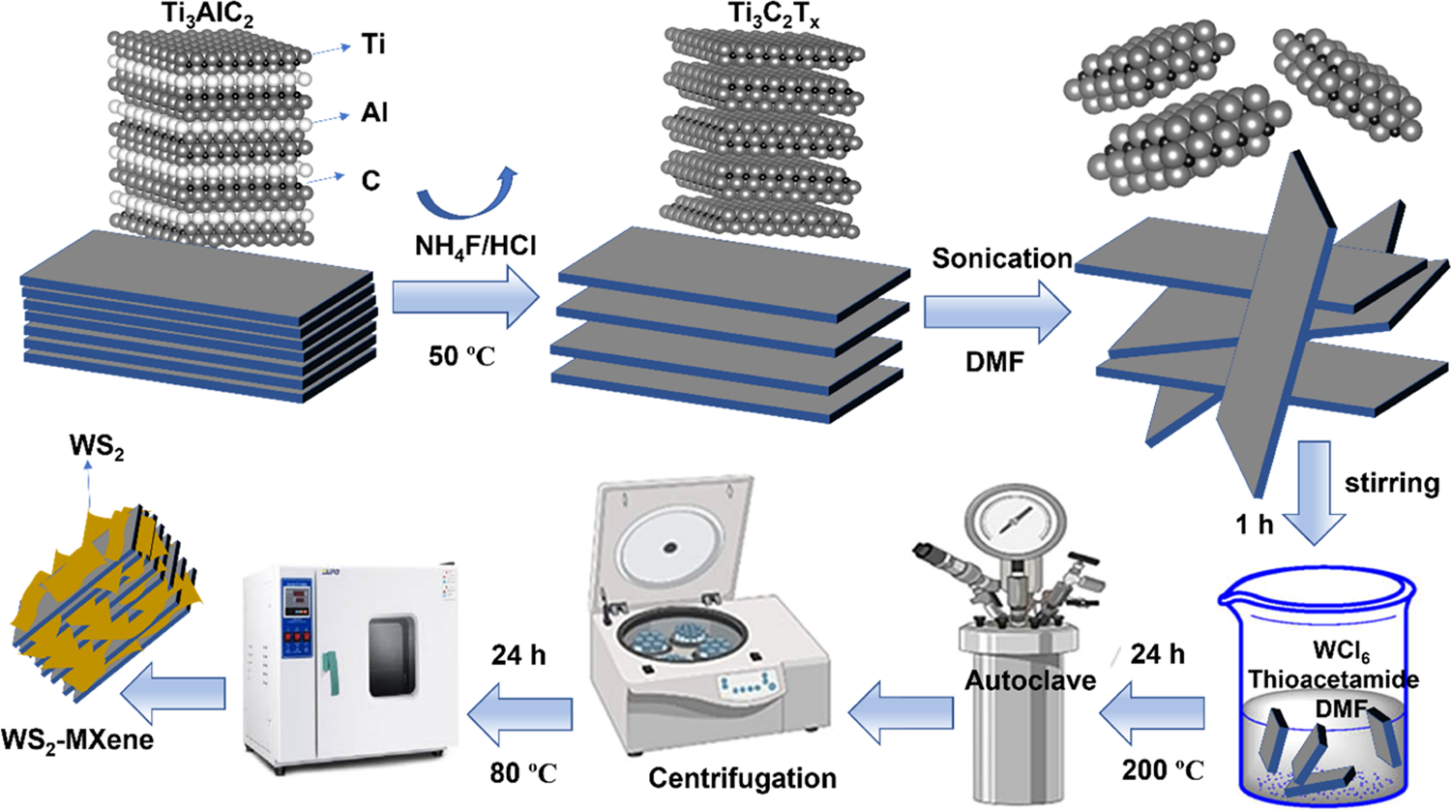
Diagrammatic Illustration Showing Development of a 2D–2D WS_2_–MXene Heterostructure through a One-Step Solvothermal Reaction

The WS_2_–Ti_3_C_2_T_*x*_ 2D–2D nanocomposite electrocatalyst materials were synthesized by using a one-step solvothermal approach, as illustrated in Scheme 1. Briefly, we optimized the reaction conditions for the safe in situ HF formation, used for etching out of the Al layer from the MAX phase to obtain MXene (Table S1). The idea was to instigate 2D–2D heterostructure interactions between WS_2_ nanopetals as the active electrocatalyst and Ti_3_C_2_T_*x*_ MXene layers as the support for the electrocatalyst. These interactions were expected to enhance the charge transfer and reductive capabilities on the sulfur active sites of the heterostructure. Consequently, a series of WS_2_–MXene hybrid electrocatalysts were prepared with varying loads of the active phase (WS_2_). Additionally, pure WS_2_ nanopetal structures were synthesized using the solvothermal method under similar conditions for the purpose of comparison.

**Figure 1:**
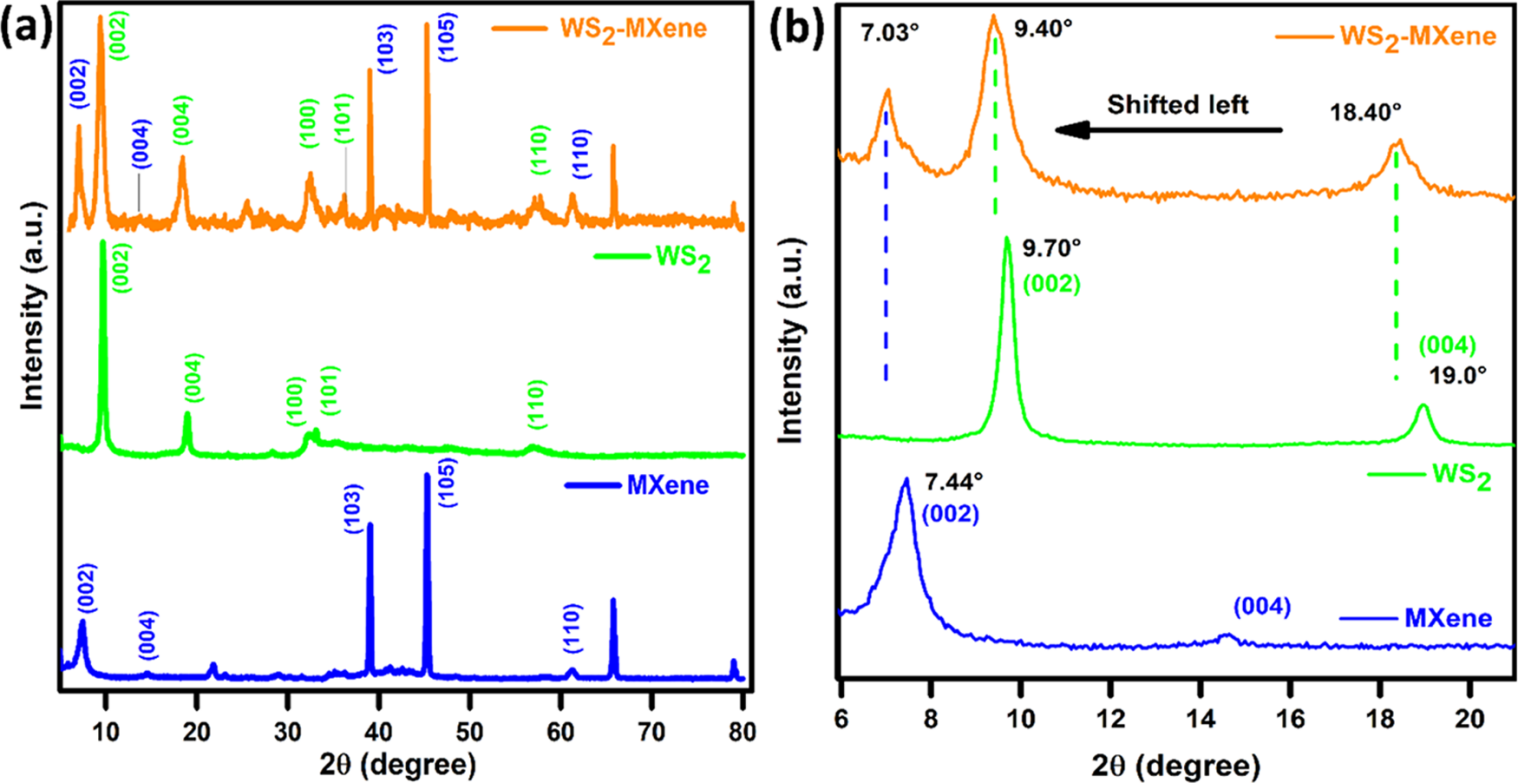
XRD pattern of (a) MXene, WS_2_, and WS_2_–MXene, (b) showing the shift of peaks in the hybrid compared with individual constituents.

The crystal structure and phases present in the samples were analyzed using the powder XRD technique. As shown in Figure 1a, the XRD patterns for the pristine Ti_3_C_2_T_*x*_ MXene, pure WS_2_, and WS_2_–MXene heterostructures were compared. For pure WS_2_, the observed peaks for the (002), (004), (100), (101), and (110) planes correspond to the hexagonal structure of WS_2_, indicating the formation of WS_2_ nanosheets that correspond well with the JCPDS card no: 08–0237.^27^ No additional peaks were detected, confirming the formation of WS_2_ nanosheets in their purest form. Furthermore, Ti_3_C_2_T_*x*_ MXene formation was confirmed by the shift of the peak at 9.20° corresponding to the plane (002) in the MAX phase to a lower 2θ value of 7.41° (Figure S1).^28^ We also found the *d*-spacing of the (002) plane in the MAX phase and MXene using eq S1. The interlayer spacing between the stacked layers of MXene was found to be 11.92 Å, an increase of 2.156 Å from the *d*-spacing of 9.764 Å obtained in the MAX phase for the (002) plane. Broadening of the (002) peak was also observed during the transformation from the MAX phase to MXene when the Al layer was etched out. These attributes demonstrate that the MXene layers are parted away and have an enlarged basal spacing for the effective in situ growth of active WS_2_ between the MXene layers.^28^ In our synthesized hybrid WS_2_–MXene nanostructures, characteristic peaks from both individual WS_2_ and Ti_3_C_2_T_*x*_ MXene were observed. The enhanced peak intensity and a higher number of crystalline peaks indicate improved crystallinity in the nanocomposites. Interestingly, the peak corresponding to the plane (002) of the MXene and the (002) and (004) planes of WS_2_ in the WS_2_–MXene hybrid shifted to a lower 2θ value, as shown in magnified Figure 1b. This can be attributed to the intercalation of WS_2_ nanopetals into the Ti_3_C_2_T_*x*_ MXene layers. These changes may be further attributed to the introduction of WS_2_ nanopetals in between the MXene layers, further parting away the MXene layers, which leads to the variation in the lattice parameters.^29^

**Figure 2:**
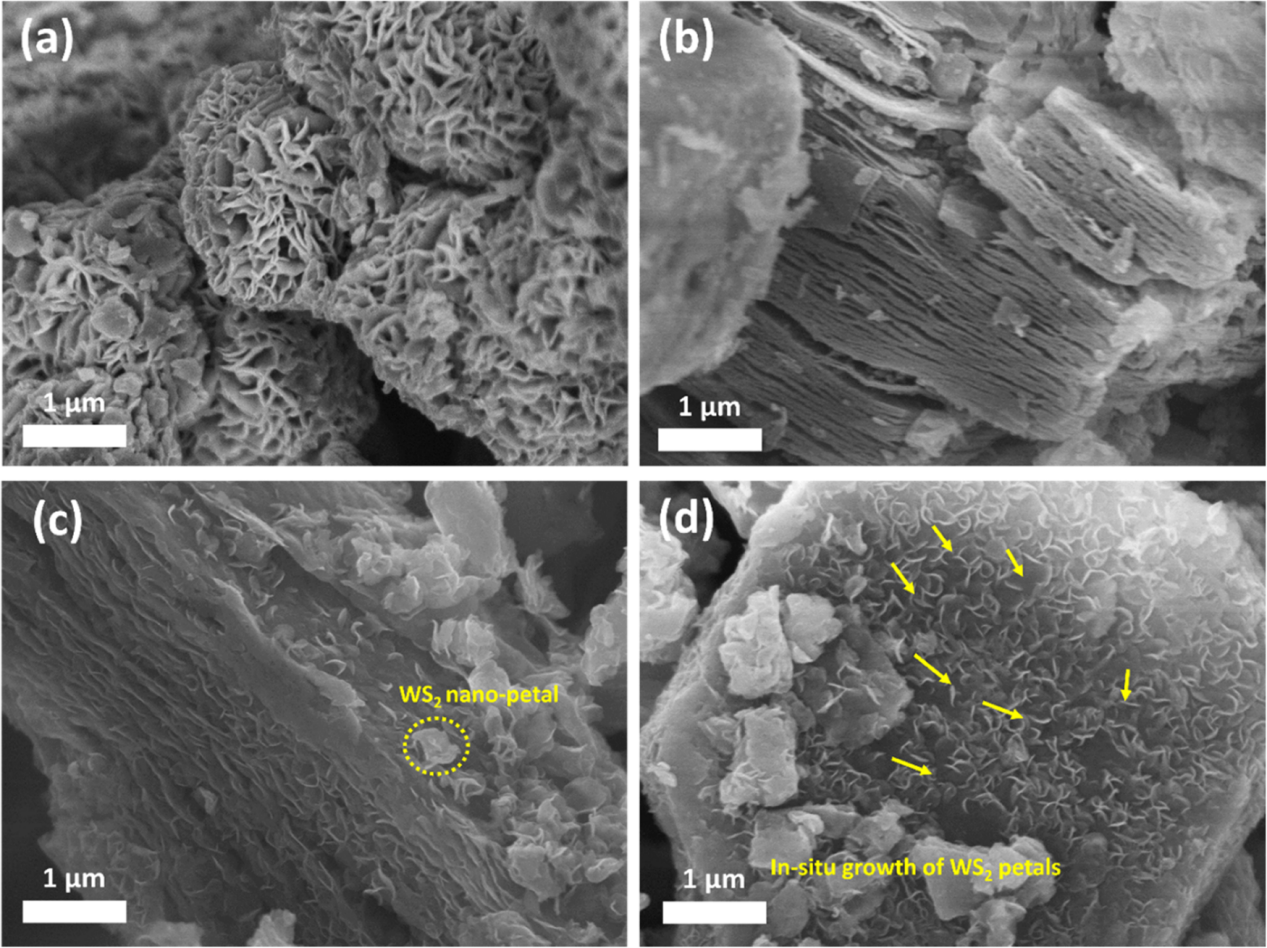
SEM images taken at a 1 μm scale for (a) pristine WS_2_, (b) pure exfoliated MXene, and (c, d) 2D–2D WS_2_–MXene nanocomposite.

The microscopic structure and morphology of the synthesized electrocatalysts were determined using scanning electron microscopy (SEM). The morphology of three nanomaterials, namely, pure WS_2_, pristine Ti_3_C_2_T_*x*_ MXene, and a nanocomposite combination of 5% WS_2_–MXene, was analyzed. The micrograph for pristine WS_2_ (Figure 2a) indicates a flower-like morphology consisting of numerous individual nanopetal-shaped flakes that are loosely arranged together, therefore forming tightly packed flowers with many voids. In contrast, the MXene micrograph depicts an accordion-like morphology, as depicted in Figure 2b, where the Ti_3_C_2_T_*x*_ MXene layers can be seen parted away from each other, like loosely held pages of a book. The cross-sectional view of the 5% WS_2_–MXene heterostructure sample can be seen in Figure 2c,d, depicting the uniform dispersion and inclusion of WS_2_ nanopetals both on plane-view and interlayer spaces of the MXene layers, resulting in wrinkled nanosheets of the 5% WS_2_–MXene heterostructure electrocatalyst.

**Figure 3:**
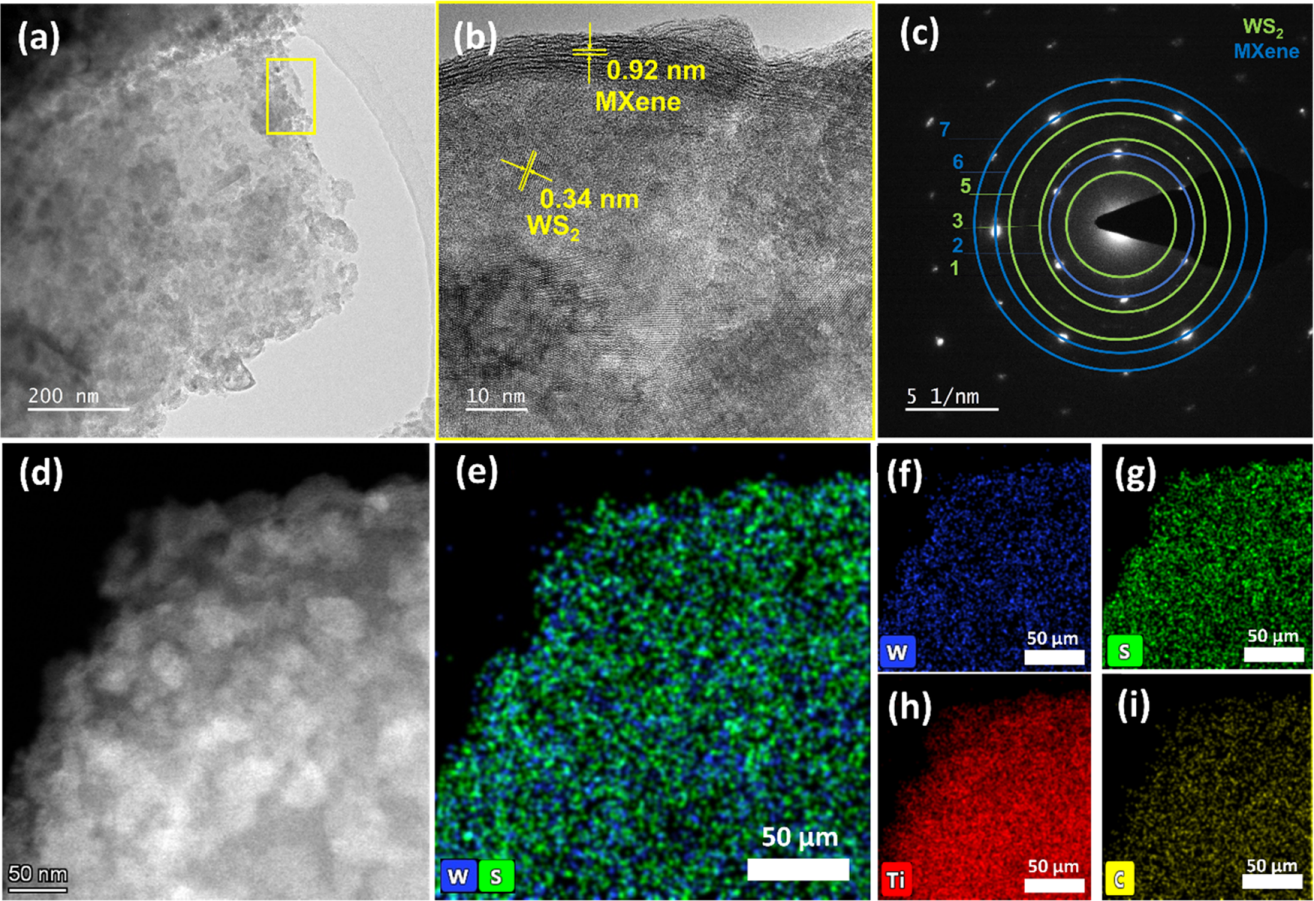
(a) TEM image, (b) high-resolution TEM image, (c) SAED, (d) HAADF-STEM, and elemental mapping showing the (e) W and S, (f) W, (g) S, (h) Ti, and (i) C of the 2D–2D WS_2_–MXene heterostructure.

High-resolution transmission electron microscopy (HRTEM) was also employed to analyze the lattice structure of the heterostructure at the nanoscale, as shown in Figure 3a. A cross-sectional view of MXene flakes revealed the interlayer spacing as well as the MXene layer thickness. The HRTEM image in Figure 3b shows an interlayer distance of ∼0.92 nm, which matches with the *d*-spacing of the (002) plane of the MXene, and a *d*-spacing of ∼0.34 nm, attributed to the (004) plane of WS_2_ in the 2D–2D heterostructure.^30^ In Figure 3c, the selected area electron diffraction (SAED) pattern of the 5% WS_2_–MXene nanocomposite can be seen. The diffraction rings (1,3,5) correspond to WS_2_, while the rings (2,6,7) represent MXene, as indicated in the image. To further verify the presence and distribution of these elements, we carried out the elemental mapping with X-ray dispersive spectroscopy (EDS) in high-angle annular dark-field scanning transmission electron microscopy (HAADF-STEM) imaging mode. The contrast in the form of patch-like regions that are visible in the W and S maps confirms that the white regions in Figure 3d correspond to WS_2_. Moreover, Figure 3e–i demonstrates that the elemental mapping results validate the presence and respective distribution of W, S, Ti, and C elements. All of the aforementioned characterization validates the successful construction of the 2D–2D WS_2_–MXene heterostructure.

Raman spectroscopy was employed to analyze the atomic vibrations and investigate the structural properties of the produced electrocatalysts, as depicted in Figure S2(a). In the pure Ti_3_C_2_T_*x*_ MXene, various peaks determine different vibrational modes, as reported by Sarycheva and Gogotsi.^31^ The vibrations of Ti–C and C–C atoms (A_1g_ symmetry) correspond to peaks at 150 and 208 cm^–1^, respectively, while the O atom (T_*x*_ atoms) E_g_ vibration is attributed to peaks between 230 and 470 cm^–1^. Additionally, the vibration of C atoms in A_1g_ symmetry produces a peak at 630 cm^–1^.^28,31^ The peaks observed at Raman shift values of 357.3 and 420.1 cm^–1^ are attributed to the in-plane and out-of-plane vibrations of WS_2_, respectively, as reported in a previous study by Srinivaas et al.^32^ More importantly, the J_1_, J_2_, A_g_, and J_3_ peaks signify the rich metallic 1T phase of pure WS_2_.^32,33^ However, following the successful intercalation of WS_2_ into the WS_2_–MXene heterostructure, the characteristic peaks related to WS_2_ were recorded, but the E_2g_ and A_1g_ peaks were little red-shifted to 354.5 and 419.5 cm^–1^, respectively, as can be seen in Figure S2(b).

Therefore, by aligning the majority of the peaks with those reported in the literature, the Raman spectra verify the formation of the WS_2_–MXene heterostructure. Analysis was expanded to understand various functionalities using FTIR spectroscopy, as shown in Figure S3. It was observed that the majority of peaks in the spectra correspond to the functional groups present in the heterostructure material. The combinative results of these techniques indicate the successful formation of WS_2_–Ti_3_C_2_T_*x*_ MXene nanocomposite materials.

In addition, XPS was employed to confirm the elemental composition and valence oxidation states of the constituent atoms in pure Ti_3_C_2_T_*x*_ MXene, WS_2_, and 5% WS_2_-MXene nanocomposite. The survey spectrum for all three samples is provided in Figure S4, which exhibits peaks for all of the constituent elements with no evident reflections for any impurities. High-resolution core-level spectra were obtained through deconvolution, providing information on valence oxidation states. All of the spectra were corrected and calibrated as per C 1s standard peak at 284.8 eV. For pure MXene, the high-resolution spectra for the individual elements are provided in Figure S5 in the Supporting Information, which again confirms the formation of pure Ti_3_C_2_T_*x*_ MXene after successful etching out of the Al layer.

**Figure 4:**
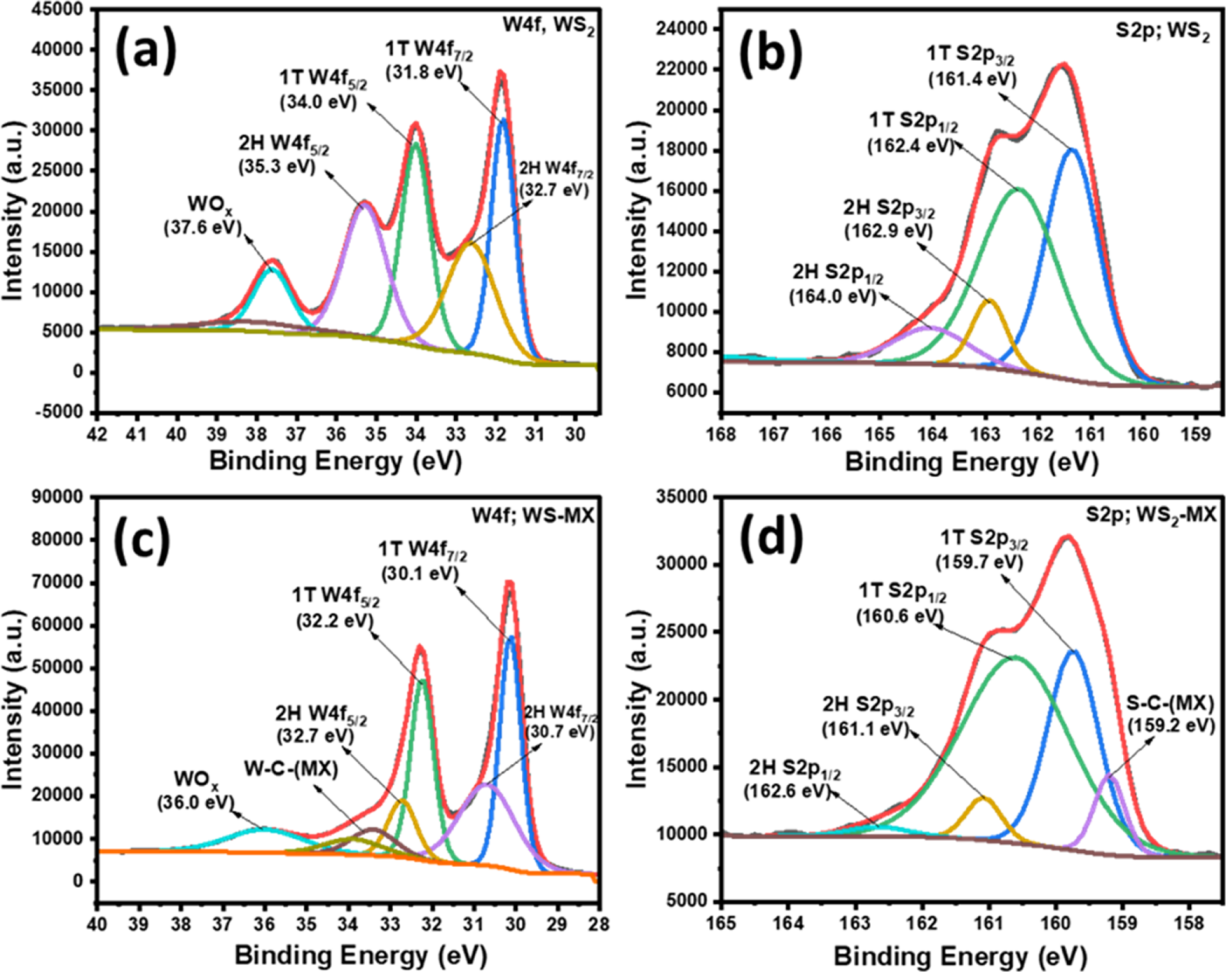
High-resolution XPS spectra of (a) W 4f of WS_2_, (b) S 2p of WS_2_, (c) W 4f of 5% WS_2_–MXene, and (d) S 2p of 5% WS_2_–MXene.

For WS_2_, the survey spectrum in Figure S4(b)confirms the formation of pure WS_2_. The S/W atomic ratio equals ∼2.17, which indicates a nearly stoichiometric composition of WS_2_. The high-resolution W 4f spectrum presented in Figure 4a exhibits a total of four peaks, which were deconvoluted into five peaks. Among these, the reflections at 31.8 and 34.0 eV were attributed to W 4f_7/2_ and W 4f_5/2_, respectively, which correspond to the 1T phase of WS_2_, while the other two peaks at 32.7 and 35.3 eV were attributed to the respective 2H phase.^23,34^ Further, a smaller peak at 37.6 eV was observed, corresponding to W^6+^ oxide.^34^ While integrating the total area of 1T and 2H phase peaks, the 1T phase was found to be 50.2%, while 2H was 49.8% in pure WS_2_. Similarly, in the S 2p core spectrum (Figure 4b), we observed two strong peaks at 161.4 and 162.4 eV, corresponding to S 2p_3/2_ and S 2p_1/2_, respectively, which are attributed to the 1T-WS_2_ phase, while two smaller peaks at 162.9 and 164.0 eV were attributed to 2H-WS_2_.^34^ Furthermore, we analyzed the high-resolution spectrum for the 5% WS_2_–MXene sample. The W 4f spectrum of the 5% WS_2_–MXene sample exhibited two major characteristic peaks with one small oxide peak. The peaks were deconvoluted into various reflections corresponding to different phases and electronic states of W 4f in the composite (Figure 4c). The two major peaks at 30.1 and 32.2 eV were attributed to the 1T phase of WS_2_, while two smaller peaks at 30.7 and 32.7 eV correspond to the respective 2H phase of W 4f_7/2_ and W 4f_5/2_, respectively. Further, two weaker peaks were observed at 33.5 and 33.9 eV, which can be assigned to W–C and W–F interactions in the nanocomposite sample, respectively.^33^ The S/W atomic ratio in the 5% WS_2_–MXene sample was found to be ∼2.08, which again confirms the intact stoichiometry of WS_2_ in the composite. However, the integrated peak areas of different W 4f phases reveal that the 1T phase and 2H phases are 51.75 and 48.25%, respectively, which suggests slight dominance of the 1T phase in the 5% WS_2_–MXene sample. This suggests that the in situ reaction between the constituent moieties for the nanocomposite formation has undergone significant interactions that slightly altered the phase proportions between 1T and 2H-WS_2_. The S 2p high-resolution spectrum in the 5% WS_2_–MXene sample showed a characteristic broad peak with a shoulder, which was deconvoluted into five different peaks (Figure 4d). Similar to pure WS_2_, the two big peaks at 159.7 and 160.6 eV were attributed to 1T-WS_2_, while peaks at 161.1 and 162.7 eV were assigned to 2H-WS_2_. Another prominent peak at 159.2 eV was assigned to the S–C–(MXene), which again confirms the robust interactions between WS_2_ and Ti_3_C_2_T_*x*_ in the nanocomposite.^35^ The S/W stoichiometric ratios for pure WS_2_ and 5% WS_2_–MXene were found to be ∼2.17 and ∼2.08, respectively (Table S2), indicating that S vacancies (if any) were slightly enhanced during the in situ nanocomposite formation. This may have helped in the HER activity, as revealed by the theoretical DFT analysis discussed in the below sections.^36^ Further, a significant shift of 1–2 eV toward lower binding energy was observed, both in the W 4f and S 2p peaks, from pure WS_2_ to the 5% WS_2_–MXene composite. This indicates effective interactions between WS_2_ and Ti_3_C_2_T_*x*_ moieties and reveals charge transfer from MXene toward WS_2_ for the effective HER.^6^ The core-level spectra of Ti 2p, C 1s, O 1s, and F 1s for 5% WS_2_–MXene are also provided in Figure S5(a–d) in the Supporting Information, which highlights the various interactions between the constituent moieties in the 5% WS_2_–MXene nanocomposite.^37^

**Figure 5:**
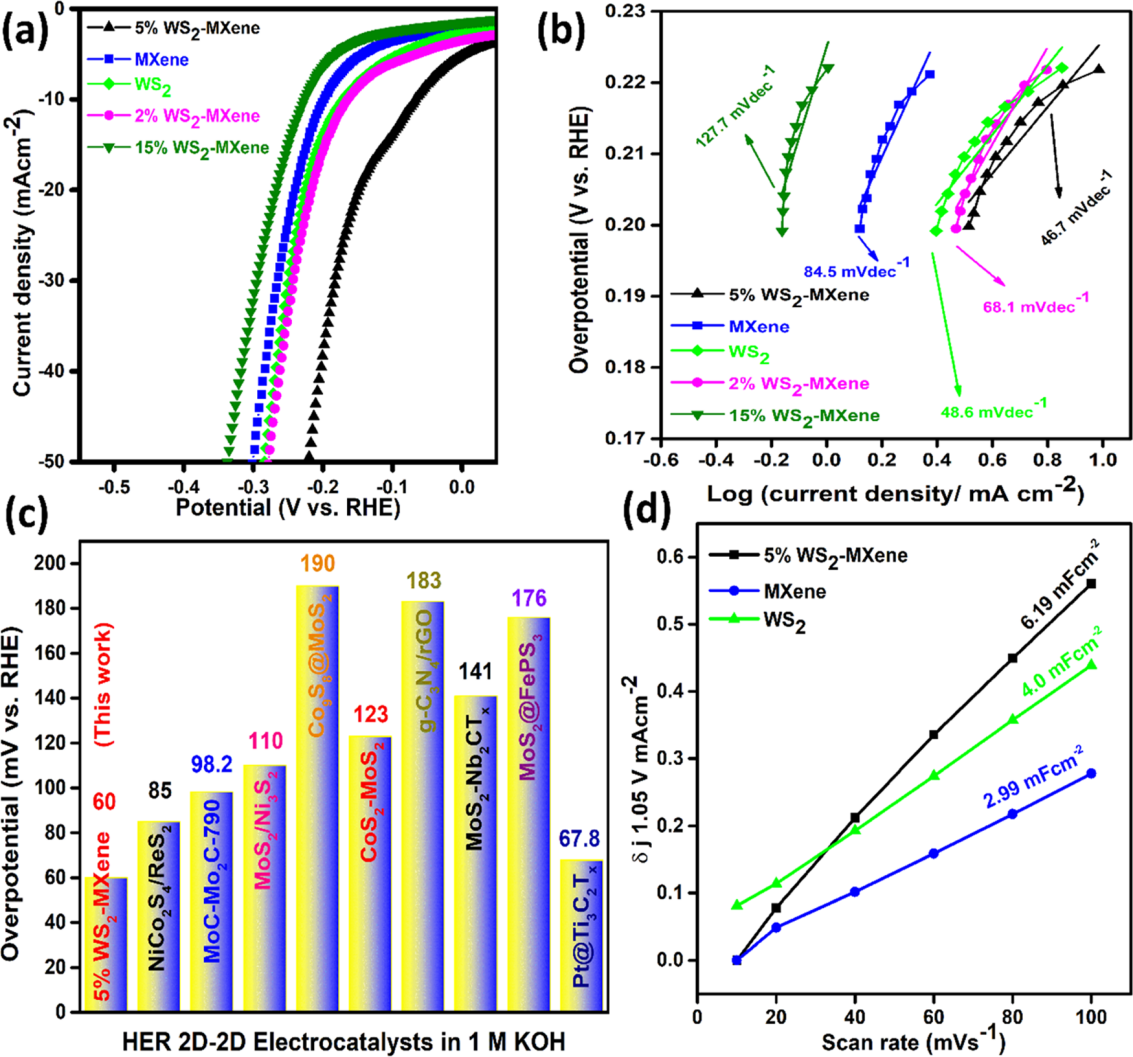
(a) Polarization curves and (b) Tafel plots of 5% WS_2_–MXene. (c) Bar chart showing the overpotential at −10 mA cm^–2^ for 2D–2D electrocatalysts’ comparison from previous work and this study in 1 M KOH. (d) Linear relationship between the scan rate and capacitive current density.

After the successful development of the 2D–2D nanocomposite between WS_2_ and Ti_3_C_2_T_*x*_ MXene, these hybrid nanomaterial electrocatalysts were employed for electrocatalytic water splitting to generate hydrogen, using a well-equipped three-electrode setup. The HER performance of the as-prepared samples was evaluated by depositing them on NF, which was then utilized as a working electrode. LSV plots were recorded in 1 M KOH as the electrolyte, and the results are presented in Figure 5a. From the polarization plots, it is evident that 5% WS_2_–MXene demonstrated a remarkably low overpotential (η_10_) of 66.0 mV at a current density of −10 mA cm^–2^, outperforming pure WS_2_ (η_10_ = 170 mV), pristine MXene (η_10_ = 198 mV), 2% WS_2_–MXene (η_10_ = 164 mV), and 15% WS_2_–MXene (η_10_ = 227.1 mV) electrodes. This 5% WS_2_–MXene also exhibited low overpotentials (212, 270, 325, 370, 414, and 460 mV) even at higher current densities (−50, −100, −150, −200, −250, and −300 mA cm^–2^), as shown in Figure S7. The superior activity in 5% WS_2_–MXene can be attributed to the appropriate amount of the active phase WS_2_, which resulted in an efficient heterostructure with a well-distributed active site. As mentioned earlier, the appropriate distribution and dominant embedment of WS_2_ nanopetals over the basal planes of MXene and at the edges provided robust interfaces, thereby providing efficient electronic coupling that boosts the charge transfer.^38^ The loosely stacked layers of the MXene in the hybrid electrocatalysts were activated with the exposed sulfur atoms from WS_2_, which generated ample active sites for H adsorption. This was supported by the enhanced ECSA obtained in the case of 5% WS_2_–Ti_3_C_2_T_*x*_ MXene.^39^ These active sites favored H^+^ chemisorption and promoted efficient charge transfer for H_2_O reduction. This indicates that the pristine MXene and WS_2_ basal planes had relatively inactive sites for H_2_ generation.^40^ The interfacial integration between WS_2_ nanopetals and the Ti_3_C_2_T_*x*_ MXene layers contributed to synergistic phenomena, proving to be the key factor in the high electrocatalytic activity toward HER.^41^ Moreover, these attributes favor 5% WS_2_-MXene as a suitable candidate, achieving activity close to state-of-the-art for overall water splitting in an alkaline medium, as previously reported by 2D electrocatalysts.^42^

Among the electrochemical parameters considered in evaluating the activity of an electrocatalyst, the Tafel slope is very significant, as it determines the kinetics of the HER process.^41,43,44^ The Tafel plots obtained for the synthesized electrocatalyst are shown in Figure 5b. The Tafel slope values were obtained for pure MXene (84.6 mV·dec^–1^), pure WS_2_ (48.5 mV·dec^–1^), 2% WS_2_–Ti_3_C_2_T_*x*_ (68.1 mV·dec^–1^), 5% WS_2_–Ti_3_C_2_T_*x*_ (46.7 mV·dec^–1^), and 15% WS_2_–Ti_3_C_2_T_*x*_ (127.7 mV·dec^–1^). Among the synthesized electrocatalysts, 5% WS_2_–MXene showed the lowest Tafel slope value of 46.7 mV·dec^–1^, which was closest to that of Pt/C, indicating the fastest electron-transfer kinetics and better HER activity.^45^Figure 5c provides a comparison between the HER performance of 5% WS_2_–MXene and previously reported 2D–2D electrocatalysts for HER in 1 M KOH.^46−53^

Broadly, the process of alkaline HER consists of two steps. The first step is expressed by two mechanistic routes, known as Volmer and Heyrovsky, that are represented by eqs 2 and 3, respectively, whereas the second step is shown by eq 4 that represents the Tafel reaction for HER.^38^
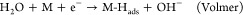
2
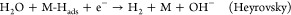
3
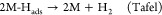
4

Here, symbols “M” and “M-H_ads_” represent an exposed hybrid surface that participates in HER and the intermediate hydrogen species adsorbed on the surface, respectively. By examining the Tafel slope, it is possible to identify the step that limits the rate of HER. The Butler–Volmer kinetics proposes that if the Tafel slope values for the Tafel, Heyrovsky, or Volmer steps are 30, 40, and 120 mV, respectively, then the rate-determining step for alkaline HER can be determined.^54^ In this study, the most active electrocatalyst, 5% WS_2_–MXene, follows Heyrovsky as the rate-determining step for the HER, which indicates that more protons are being adsorbed on the electrocatalyst surface.

In order to better understand the best electrocatalyst (5% WS_2_–MXene) that showed the best activity with respect to its other counterparts, electrochemical surface area (ECSA) and double-layer capacitance (*C*_dl_) were determined.^55^ Cyclic voltammetry (CV) in the nonfaradaic region was employed in Figure S(8)at various scan rates ranging from 10 to 100 mV s^–1^. The calculated *C*_dl_ values of 5% WS_2_–MXene, MXene, and WS_2_ are presented in Figure 5d. The results show that 5% WS_2_–MXene has the highest *C*_dl_ value of 6.19 mFcm^–2^, indicating that the well-supported features of the MXene multilayers possibly ensure the well-dispersed WS_2_ nanopetals. Consequently, there is a higher buildup of charge between the electrode and the electrolyte region, which enhances the charge transfer capability of the electrocatalyst.^56^ Alongside, the characteristic of the hybrid is also the reason for enhanced charge transfer across the interface compared to their individual counterparts. Therefore, a high *C*_dl_ value signifies high HER activity.^43,57^

**Figure 6:**
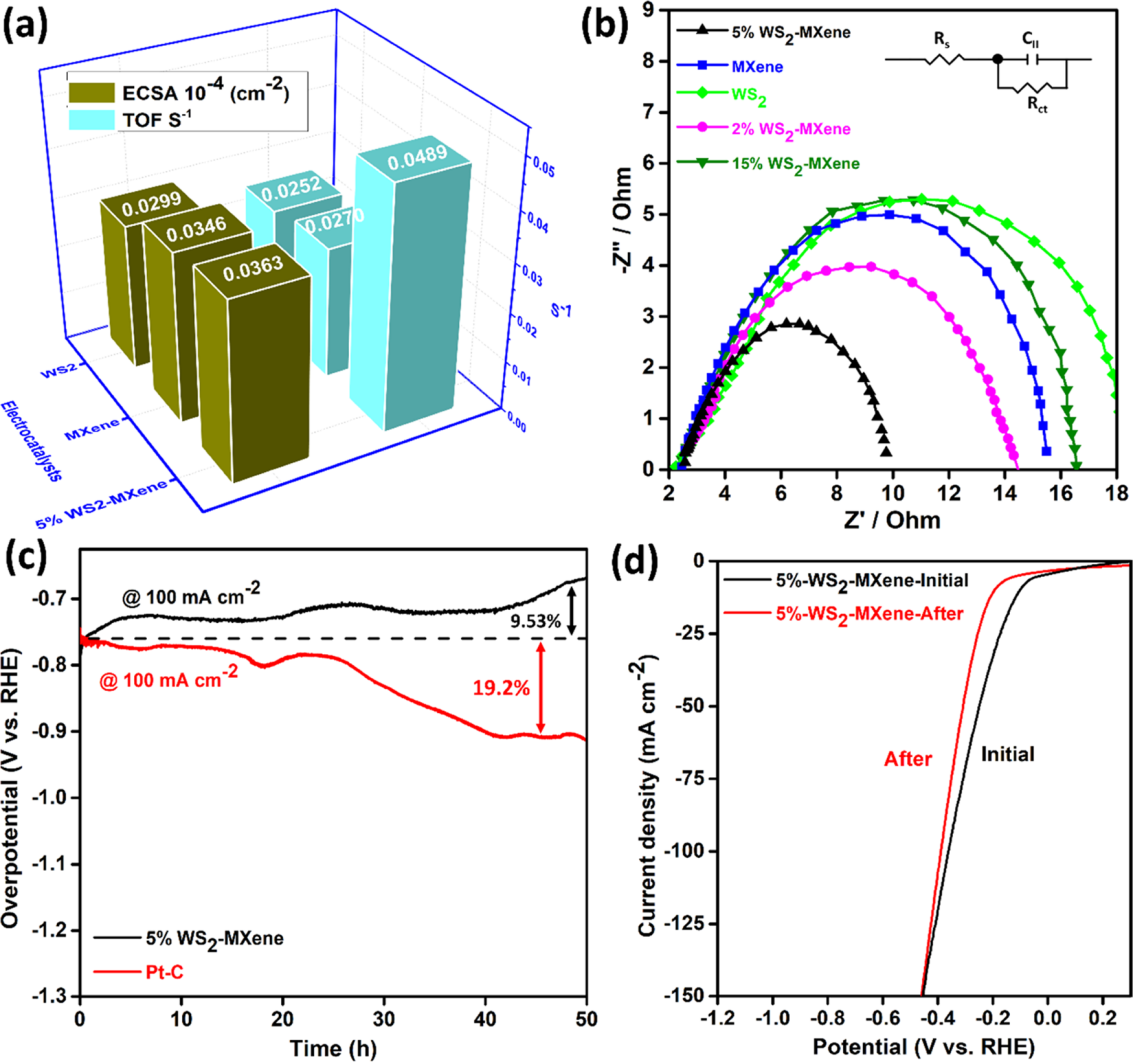
(a) Comparison of ECSA and TOF for 5% WS_2_–MXene, MXene, and WS_2_. (b) EIS Nyquist plot and (c) stability of 5% WS_2_–MXene in comparison to pure WS_2_ and MXene for 12 h, where the inset shows the zoom-in trend of WS_2_ and MXene. (d) Stability of 5% WS_2_-MXene for 50 h.

In addition, the values for ECSA and TOF were calculated (details in the Supporting Information) and were found to be (3.63, 3.46, and 2.99) × 10^–6^ cm^–2^ and (0.0489, 0.0270, and 0.0252) S^–1^, respectively, for 5% WS_2_-MXene, MXene, and WS_2_, as depicted in Figure 6a. The active site density calculated in the process (details in the Supporting Information) demonstrated that 5% WS_2_–MXene has potentially more active site density and a larger ECSA than pure MXene and WS_2_, leading to its superior HER activity. The active site density (*n*) was found to be equal to 1.05 × 10^–5^, 5.33 × 10^–6,^ and 8.23 × 10^–6^ mol cm^–2^ for 5% WS_2_–MXene, pure MXene, and WS_2_ samples, respectively. Also, 5% WS_2_–MXene displayed the highest TOF, which indicated more catalytic reactions on the active sites per unit time. Therefore, the superior HER activity in 5% WS_2_–MXene was attributed to larger ECSA, higher active site density, and enhanced charge transfer, as indicated by the EIS study discussed below.

In order to test the efficacy of electrocatalysts in transferring charge across the electrode–electrolyte interface, EIS was performed in a 1 M KOH electrolytic solution.^41,43^Figure 6b portrays the Nyquist plots obtained from EIS for the WS_2_–MXene series, along with pristine MXene and WS_2_. The Nyquist plots exhibit semicircle-type curves, where the diameter signifies the impedance to charge transfer (*R*_ct_). It can be seen that among the nanocomposite samples, the smallest semicircle diameter was obtained for 5% WS_2_–MXene, with an *R*_ct_ value of 2.56 Ω, indicating its better charge transfer due to the most compatible synergism between WS_2_ (4.07 Ω) and MXene (2.60 Ω), as shown in Table S3.^38^ When other WS_2_–MXene samples were tested, they exhibited higher *R*_ct_ values compared to 5% WS_2_–MXene. Consequently, the 5% WS_2_–MXene heterostructure electrocatalyst demonstrated lower electronic as well as ionic resistance and promoted faster electron charge transfer, thereby enhancing the electrochemical kinetics.^58^ Moreover, the better crystallinity of the composite is another factor for fast charge transfer across the electrode–electrolyte interface, outperforming their pristine counterparts.^59^

Apart from the robust activity demonstrated by the 5% WS_2_–MXene heterostructure, long-term stability is also very critical for the economic viability of the electrocatalyst. Chronopotentiometry tests were performed at constant current densities of 10, 50, and 100 mA cm^–2^ for 5% WS_2_–MXene and compared with Pt/C, as shown in Figure S10. These tests were conducted over 50 h in a 1 M KOH electrolyte. It was noticed that only a small variation in the overpotential occurred for 5% WS_2_–MXene at the various current densities over a period of 50 h of HER. When the Pt/C electrocatalyst was employed for the chronopotentiometric stability test at 100 mA cm^–2^, it exhibited a recognizably higher increase in the overpotential over a period of 50 h with respect to the 5% WS_2_–MXene sample, as illustrated in Figure 6c. Contrarily, the 5% WS_2_–MXene electrocatalyst exhibited nearly a stable overpotential for a period of approximately 20 h, which then started gradually decreasing owing to the exposure of active sites on H_2_ evolution at a higher current density of 100 mA cm^–2^. Additionally, the LSV polarization curves shown in Figure 6d demonstrate a very small increase in overpotential at 100 mA cm^–2^ after a 50 h durability test, which again suggests that the 5% WS_2_–MXene electrocatalyst is a very competitive prospect for HER with respect to the state-of-the-art Pt/C electrocatalyst. Similarly, a chronoamperometric durability test of pristine WS_2_, MXene, and 5% WS_2_–MXene for 12 h of HER was implemented, as shown in Figure S11. The higher stability recorded for the 5% WS_2_–MXene electrocatalyst can be attributed to the following two reasons: (1) the in situ synthesis resulted in effective intercalation between WS_2_ and MXene^60^ and (2) strong metal–support interaction and coupling effect between WS_2_ and MXene (as also confirmed from XPS analysis).^60^ The results from XRD, SEM, HRTEM, and EDX analysis prove that the proposed electrocatalyst (5% WS_2_–MXene) maintains its crystalline phases and morphology even after 50 h of the durability test (Figures S12 and S13). In summary, due to interfacial engineering through a single-step solvothermal synthesis, the 5% WS_2_–MXene 2D–2D heterostructure exhibited excellent HER performance in a basic medium.

To validate the utmost improvement in the HER catalytic activity for the hybrid structure considered above, DFT calculations on WS_2_–Ti_3_C_2_F_2_ were performed. The model of the hybrid structure (WS_2_–Ti_3_C_2_F_2_) was assembled according to the XPS results. Due to the high chemical activity of MXene, it is easily passivated by functional groups (–O, –OH, or –F).^61^ MXene functionalized with F was used for the calculation due to the presence of the F signal in XPS. WS_2_ dominantly forms in two phases 2H and 1T, and the 1T phase is thermodynamically more stable compared with other phases.^5^ However, from the XPS analysis of the 5% WS_2_–MXene electrocatalyst, the 1T phase was slightly dominant over the 2H phase. So, we proceed with the DFT analysis involving both the 1T- and 2H-WS_2_ phases to undergo the comparative study for the HER over the 5% WS_2_–MXene sample.

**Figure 7:**
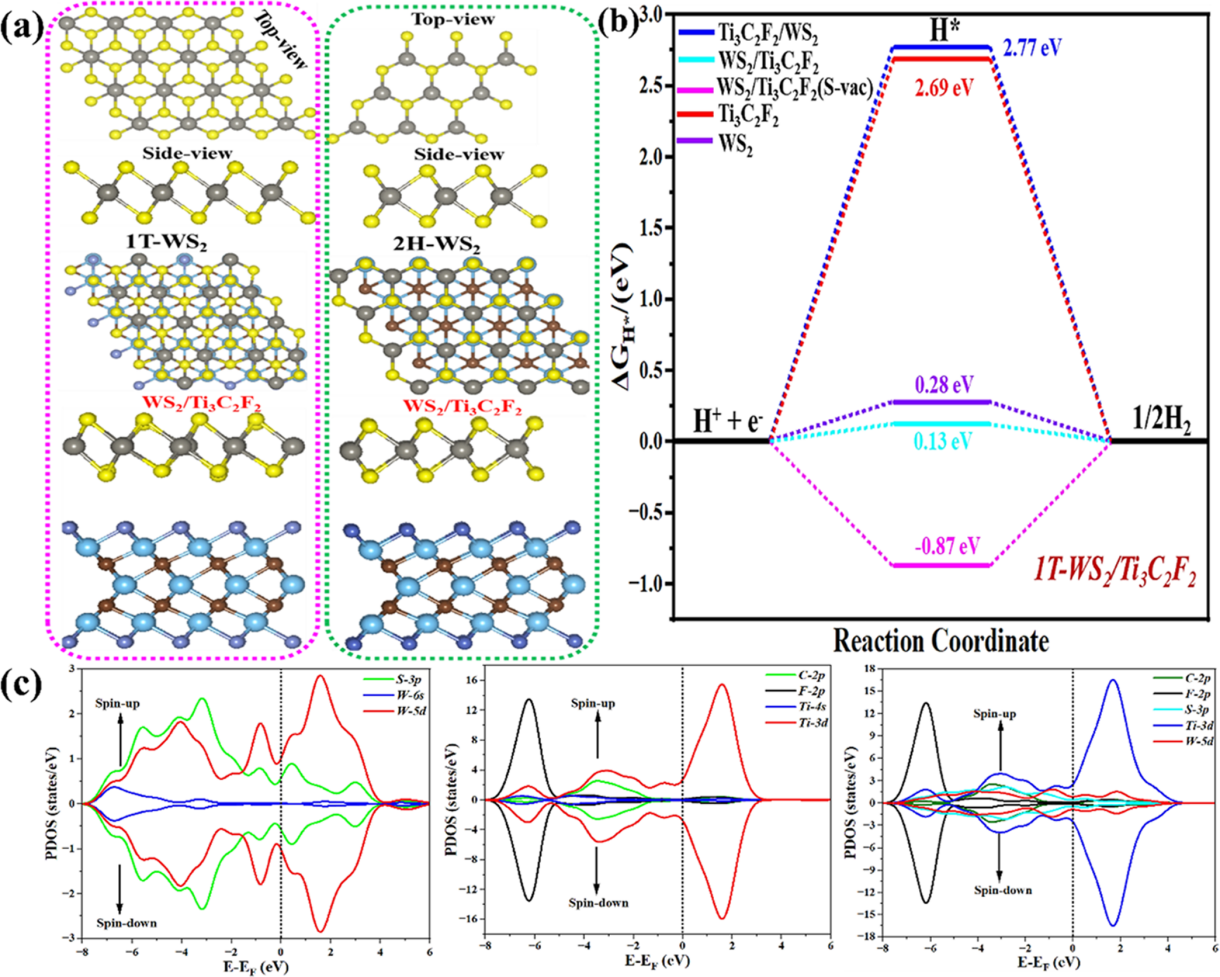
(a) Top and side views of the optimized configuration of 1T and 2H phases of WS_2_ and WS_2_–Ti_3_C_2_T_2_ surfaces. (b) The corresponding Gibbs free energy profile (Δ*G*_H*_) for HER at the active site on the WS_2_, Ti_3_C_2_T_2_, WS_2_–Ti_3_C_2_T_2_, Ti_3_C_2_T_2_/WS_2_, and WS_2_–Ti_3_C_2_T_2_(S-vac) surfaces. The absolute value of Δ*G*_H*_ for HER activity is close to zero (Δ*G*_H*_ → 0). (c) Projected DOS of the hybrid structure with individual atom contribution.

First, we calculated the structural properties of isolated monolayers of Ti_3_C_2_F_2_, and WS_2_–Ti_3_C_2_F_2_ and WS_2_ have lattice constants of 3.18 and 3.05 Å, respectively, which are in good agreement with previous reports.^62^ Such a small lattice mismatch between Ti_3_C_2_F_2_ and WS_2_ materials is acceptable and allows the construction of a heterostructure with precise stacking. The first hybrid structure was constructed by assembling 1T-WS_2_ (3.18 Å) and Ti_3_C_2_F_2_ (3.05 Å) in a 2 × 2 supercell. Figure 7a illustrates the optimized configurations of 1T- and 2H-WS_2_ and WS_2_–Ti_3_C_2_F_2_ with a lattice constant of 6.37 Å. The following discussion incorporates the stable structure of 1T-WS_2_–Ti_3_C_2_F_2_.

For HER performance on 1T-WS_2_, Ti_3_C_2_F_2_, and hybrid structure (WS_2_–Ti_3_C_2_F_2_) surfaces, first-principles-based modeling under normal reaction conditions was also carried out to understand the nature of the active site and reaction mechanism at the atomic level. The calculated Gibbs free energies for the adsorption of atomic hydrogen (G_H*_) over the WS_2_ and Ti_3_C_2_T_2_ monolayers and different combinations of the hybrid structure (MX-WS_2_-MX, MX-WS_2_-MX(F-vac), WS_2_/MX, WS_2_/MX (S-vac), MX/WS_2_, MX/WS_2_(F-vac), WS_2_-MX-WS_2_, and WS_2_-MX-WS_2_(S-vac)) at the active site have been examined, as presented in Figures 7b and S14. An optimal HER system should have Δ*G*_H*_ close to zero (Δ*G*_H*_ → 0). The result shows that the hybrid structure improves the hydrogen evolution reaction (HER) performance if H* is adsorbed on the WS_2_–Ti_3_C_2_T_2_ surface. Remarkably, the WS_2_–Ti_3_C_2_T_2_ hybrid structure exhibits outstanding catalytic activity in HER, with an optimal |Δ*G*_H*_| value of 0.13 eV, which is consistent with the experimental results in Figure 5 (Exp).

We calculated the projected density of states (PDOS) and charge density difference (CDD) of WS_2_, Ti_3_C_2_F_2_, and the hybrid structure (WS_2_–Ti_3_C_2_F_2_) to further understand the binding nature and thermodynamic stability. Figure 7b illustrates the total density of states of WS_2_, Ti_3_C_2_F_2_, and hybrid structure WS_2_–Ti_3_C_2_F_2_. We found that the total density of states for the hybrid structure WS_2_–Ti_3_C_2_F_2_ exhibits better metallic behavior in comparison to Ti_3_C_2_F_2_ and WS_2_. The presence of strong peaks near the Fermi level for both the hybrid structure and Ti_3_C_2_F_2_ specifies a high chemical reactivity, which improves HER performance. We have anticipated the spin-polarized density of states projected on the several atomic contributions (Ti 3d, C 2p, F 2p, S 3d, H 1s, and W 5d) to better understand the binding nature between the hybrid structure, as shown in Figure 7c. According to the PDOS, the major peaks of the Ti 3d orbital close to the Fermi level (*E*_F_) are evidence of the high reactivity, which might be responsible for the activation of adsorbates during catalysis reactions.^63^ Moreover, the CDDs of the different combinations of the hybrid structure WS_2_–Ti_3_C_2_F_2_ (see Figure S15) were also measured to get insights into the nature of chemical bonding between the interatomic layers. The results are presented in Figure S15. The electron density accumulation regions are rendered in red, covering the S atoms, clearly showing electron transfer from WS_2_ to Ti_3_C_2_F_2_.

The structural models of 2H-WS_2_–Ti_3_C_2_F_2_, partial density of states (PDOS), charge density difference (CDD), and work function (Φ) are presented in the Supporting Information (see Figures S16). A detailed description of these structural parameters and electronic properties is summarized in the SI (see Pages S19 and S22). Looking into the comparison, 1T-WS_2_ and 1T-WS_2_–Ti_3_C_2_F_2_ exhibit excellent HER activity with respect to 2H-WS_2_ and 2H-WS_2_–Ti_3_C_2_F_2_. Therefore, it can be concluded that more 1T-WS_2_ and Ti_3_C_2_F_2_ substrate participate in HER processes based on the electronic structure and Δ*G*_H*_ values, and the related HER processes will be greatly promoted. The aforementioned results suggest that the 1T-WS_2_–Ti_3_C_2_F_2_ hybrid structure is a promising HER model with good agreement with experimental data.

## 4 Conclusions

In summary, this work presents the development of an in situ 2D–2D nanocomposite through interactions between WS_2_ and Ti_3_C_2_T_*x*_ MXene for HER application, employing a facile single-step solvothermal technique. The petal-like WS_2_ morphologies are embedded both on and between the Ti_3_C_2_T_*x*_ MXene layers, forming a unique 2D–2D nanocomposite morphology, as confirmed by the detailed structural characterization techniques. The XPS results demonstrate the desired stoichiometry of the synthesized electrocatalysts and reveal a slight dominance of the 1T-WS_2_ phase over the 2H phase in the 5% WS_2_–MXene sample. Among all of the synthesized electrocatalysts, 5% WS_2_–MXene showed outstanding HER activity with an overpotential of 66.0 mV at −10 mA cm^–2^ and a Tafel slope of 46.7 mV dec^–1^. This exceptional performance stems from the synergistic effect between WS_2_ and Ti_3_C_2_T_*x*_ MXene, enhancing the active site density and electronic interactions. The electrocatalyst also demonstrated long-term stability (50 h) in a 1 M KOH electrolyte for HER. DFT results supported the experimental outcomes, demonstrating the lowest overpotential of 0.13 eV in 5% WS_2_–MXene, owing to the Ti 3d orbital closely aligning with the Fermi level and high metallic behavior, emphasizing charge accumulation at 1T-WS_2_ S-sites, and enhancing HER performance in terms of overpotential and Tafel slope. This study presents an innovative approach to designing cost-effective 2D–2D interfaces, opening avenues for water-splitting applications and sparking interest in future TMDC research.

## Supporting Information Available

Results such as the optimization parameters for MXene exfoliation; calculation for finding the interlayer spacing between the MAX phase and MXene before exfoliation and after etching; FTIR spectrum; XPS survey scan; SEM EDX spectrum; SEM elemental mapping; CV curves; capacitive charge determination from CV sweep; *C*_dl_, ECSA, Δ*j*, TOF, and *n* calculation was discussed; chronoamperometric and chronopotentiometric results; and DFT calculations (PDF)

## Abbreviations

HER: hydrogen evolution reaction
Pt: platinum
TMS: transition metal sulfides
H_2_: hydrogen
TMD: transition metal dichalcogenide
rGO: reduced graphene oxide
CVD: chemical vapor deposition
HDPE: high-density polyethylene
XRD: X-ray diffraction
FESEM: field emission scanning electron microscopy
EDX: energy dispersive X-ray
HRTEM: high-resolution transmission electron microscopy
SAED: selected area electron diffraction
FTIR: Fourier transform infrared
XPS: X-ray photoelectron spectroscopy
NF: nickel foam
RHE: reversible hydrogen electrode
LSV: linear sweep voltammetry
CV: cyclic voltammetry
EIS: electrochemical impedance spectroscopy
ECSA: electrochemically active surface area
TOF: turnover frequency
HAADF: high-angle annular dark field
STEM: scanning transmission electron microscopy
RDS: rate-determining step
*C*_dl_: double-layer capacitance
SEI: semiconductor electrolyte interface
*R*_b_: bulk resistance
SMSI: strong metal–support interaction
Pt: platinum
PDOS: partial density of states
CDD: charge density difference
*E*_F_: Fermi level
Φ: work function
TMDC: transition metal dichalcogenide
