# Supplementary material for: In Situ Growth of Interfacially Nanoengineered 2D–2D WS2/Ti3C2Tx MXene for the Enhanced Performance of Hydrogen Evolution Reactions
Source: ACS Appl Mater Interfaces. 2024 Mar 12;16(11):14229–42. doi: 10.1021/acsami.3c11642 (PMC10958446; doi:10.1021/acsami.3c11642)
Supplement: Supplementary file 1 — am3c11642_si_001.pdf [file am3c11642_si_001.pdf]

Supporting Information

In-situ Growth of Interfacially Nano-engineered 2D-2D $WS_2/Ti_3C_2T_x$ MXene for Enhanced Performance of Hydrogen Evolution Reactions

Faisal Rasool,[†] Bilal Masood Pirzada,[†] Shamraiz Hussain Talib,[†,] Tamador Alkhidir,[†] Dalaver H Anjum,[⊥] Sharmarke Mohamed,[†] Ahsanulhaq Qurashi[†,]*

[†] Department of Chemistry, Khalifa University of Science and Technology, Abu Dhabi 127788, United Arab Emirates

 Center for Catalysis and Separations, Khalifa University of Science and Technology, Abu Dhabi, P.O. Box 127788, United Arab Emirates

[⊥] Department of Physics, Khalifa University of Science and Technology, Abu Dhabi 127788, United Arab Emirates

Author's email address:

F.R.: 100059711@ku.ac.ae, B.M.P.: bilal.pirzada@ku.ac.ae, S.H.T.: shamraiz.talib@ku.ac.ae, T.A.: tamador.alboshra@gmail.com, D.H.A.: dalaver.anjum@ku.ac.ae, S.M.: sharmarke.mohamed@ku.ac.ae

* Corresponding author email address and phone number:
A.Q.: ahsan.qurashi@ku.ac.ae, +971 2 312 4202

KEYWORDS: *$WS_2/Ti_3C_2T_x$ MXene, 2D-2D Heterostructure, in-situ HF, Interfacial engineering, Hydrogen Evolution Reaction, DFT*

Table S1. Summary of optimization parameters used for in-situ HF formation for chemical etching of MAX phase to produce the MXene.

Exp. No.	Precursor 1	Precursor 2	Temperature (℃)	Etching time (h)	Concentration	Results (XRD)
**1**	**1g Ti_3AlC_2**	**1 g NH_4F + 20 ml HCl + 30 ml H_2O**	**35**	**24**	**5 M HCl**	**TiC**
**2**	**0.5 g Ti_3AlC_2**	**2.07 g LiF + 20 ml HCl + 30 ml H_2O**	**Room temperature**	**24**	**5 M HCl**	**(002) Peak at 9.2º**
**3**	**1 g Ti_3AlC_2**	**5.56 g NH_4F + 24.9 ml HCl + 25.1 ml H_2O**	**50**	**24**	**6 M HCl**	**(002) Peak at 7.5º**
**4**	**3.1g Ti_3AlC_2 (Scale up)**	**5.56 g NH_4F + 24.9 ml HCl + 25.1 ml H_2O**	**50**	**24**	**6 M HCl**	**(002) Peak at 7.5º**

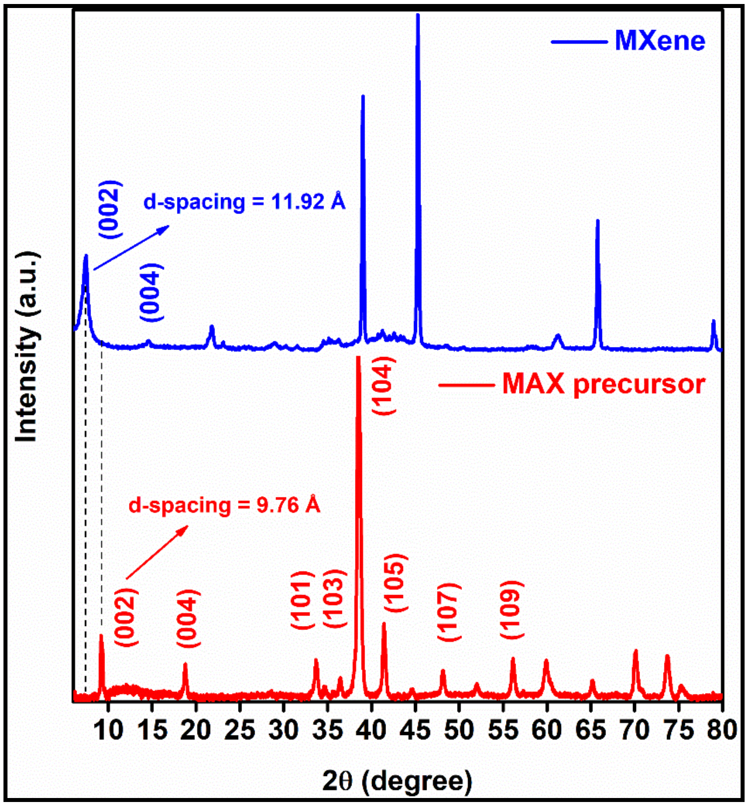

Figure S1. XRD pattern showing the MAX precursor, and MXene formed by in-situ HF formation indicating enhanced interlayer spacing of the 002 plane.

By applying Eq. S1 to the MAX phase, we calculated its d-spacing using Bragg's law. We then compared this value to the d-spacing of MXene after in-situ chemical etching, which can be observed in Figure S1.[1]

$n\,\lambda = 2\,d\sin(\theta)$ (S1)

Where,

λ = 1.5406 Å

θ = Peak position (in radians).

n = 1 (Order of diffraction).

d = Interplaner spacing or d-spacing (in Å).

<u>For MAX phase precursor 002 plane</u>

2 thetas = 9.05, theta = 4.525, d = 9.764 Å.

<u>For MXene 002 plane</u>

2 thetas = 7.41, theta = 3.705, d = 11.921 Å.

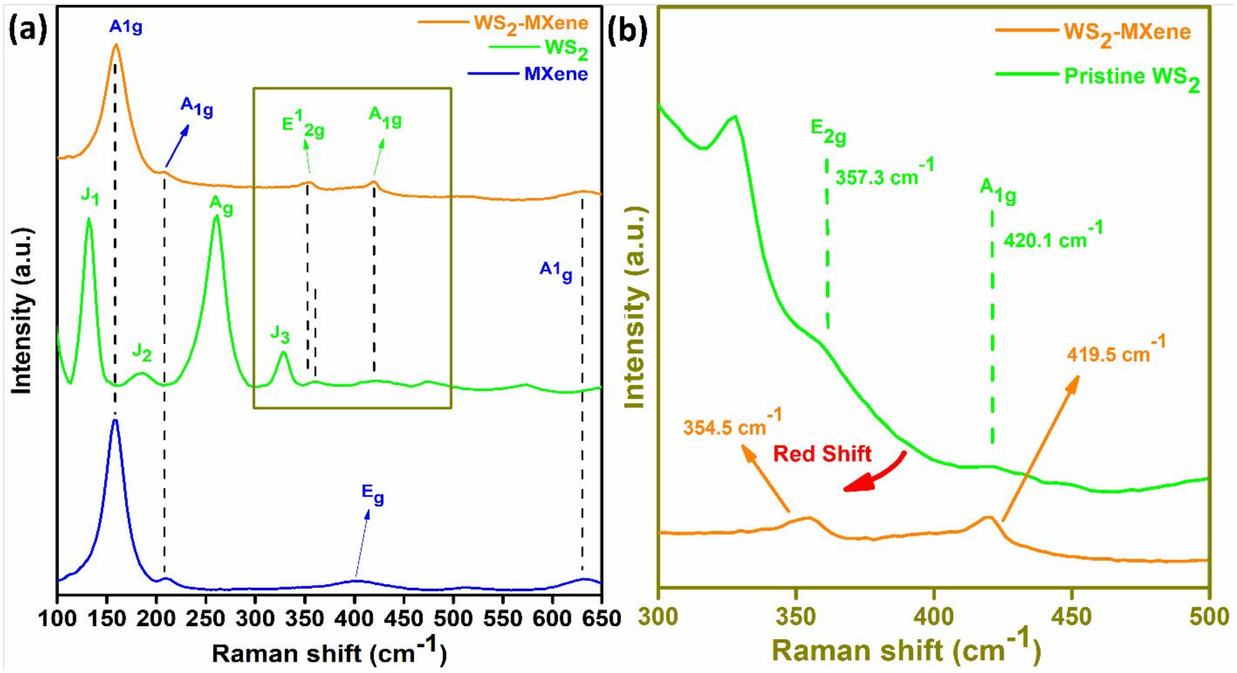

Figure S2. Raman spectra of (a) WS_2-MXene in comparison to pristine MXene, and WS_2 (b) zoomed in region showing the red shift in WS_2-MXene due to intercalation of WS_2.

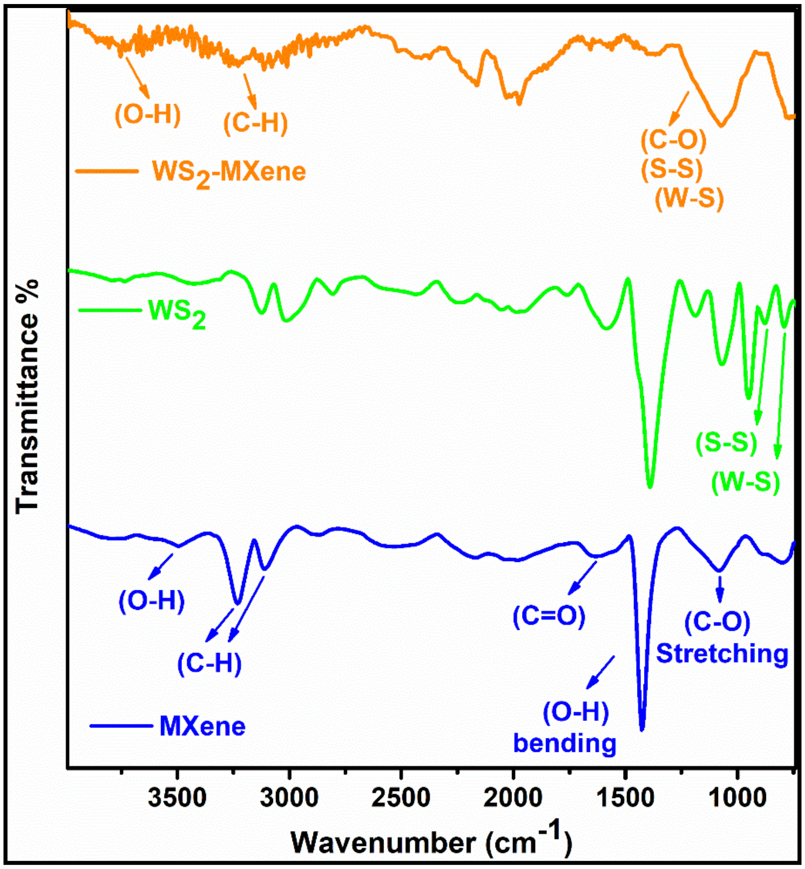

Figure S3. FTIR spectrum of pure MXene; WS_2; and 5% WS_2-$Ti_3C_2T_x$ MXene showing various functionalities present in these electrocatalysts.

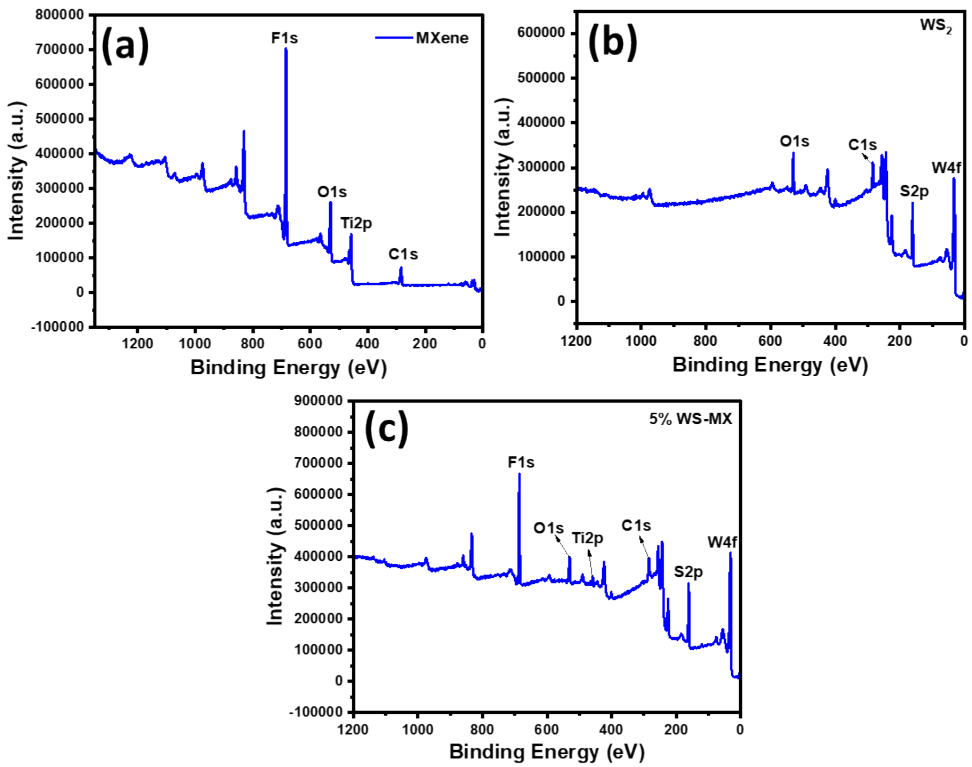

Figure S4. XPS analysis showing the survey spectrum of: (a) $Ti_3C_2T_x$ MXene; (b) WS_2 (the peaks for C and O came from the carbon tape, which was used for holding the sample); and (c) 5% WS_2-$Ti_3C_2T_x$ MXene indicates the presence of all the constituents in their desired electronic states.

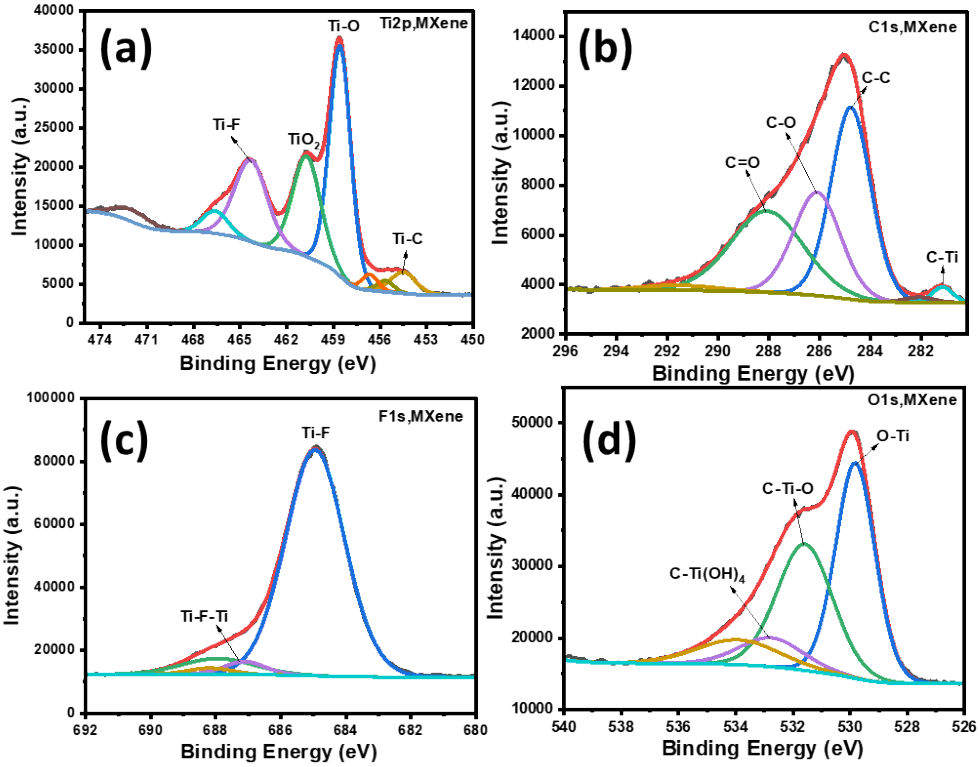

Figure S5. Core level XPS spectra of a) Ti (2p); b) C (1s); c) F (1s); and d) O (1s) in pure $Ti_3C_2T_x$ MXene.

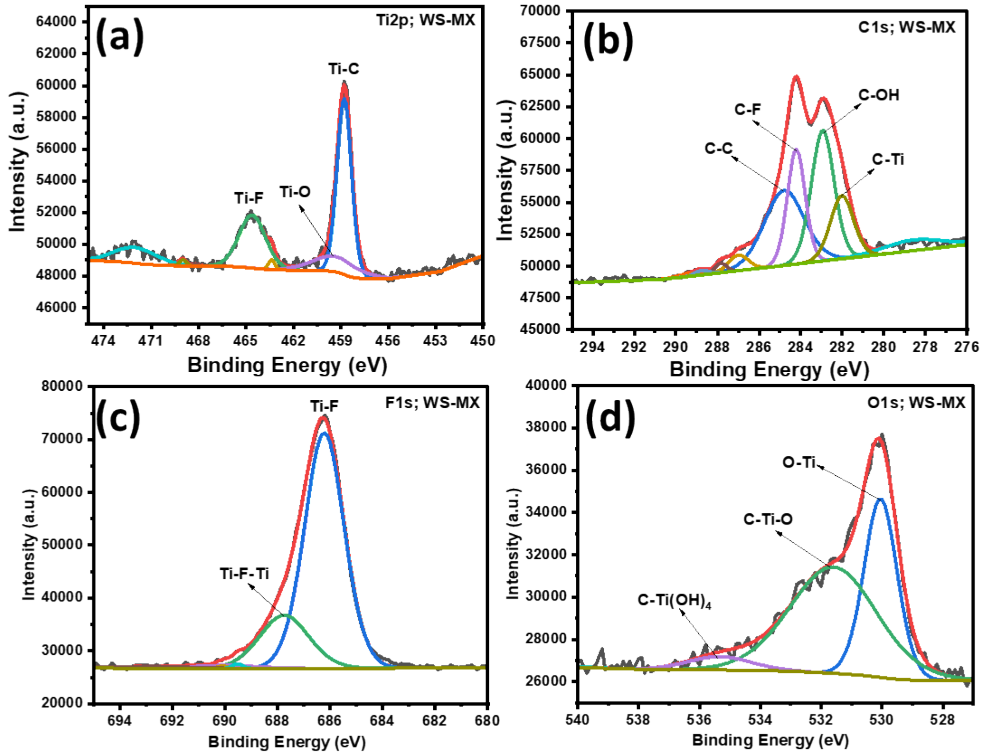

Figure S6. Core level XPS spectra of a) Ti (2p); b) C (1s); c) F(1s); and d) O (1s) in 5% WS_2-$Ti_3C_2T_x$ MXene heterostructure.

Table S2: S/W atomic ratios as obtained from the XPS analysis

Name	Peak BE	FWHM eV	Area (P) CPS.eV	Atomic %	Q
**5% WS_2-MXene**					
S2p	161.35	3.4	614767.49	23.62	1
W4f	32.47	4.57	1823542.01	11.34	1
**WS_2**					
S2p	161.48	2.19	468241.35	32.76	1
W4f	32.92	5.12	1331858.51	15.09	1

Electrochemical Measurements

The potential was converted using Eq. (S2) to a Reversible Hydrogen Electrode (RHE)

$$E(RHE) = E(Ag/AgCl) + 0.197 + 0.0591 * pH\ (14) \qquad (S2)$$

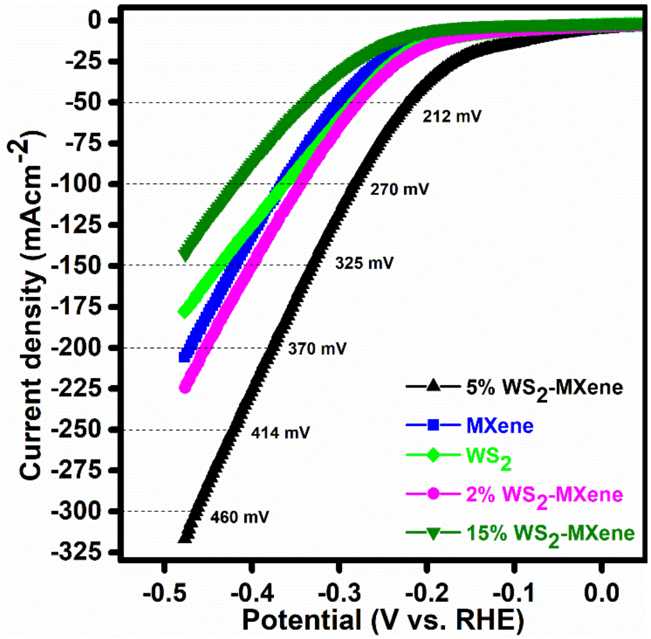

Figure S7. LSV of 5% WS_2-MXene showing overpotential at various current densities.

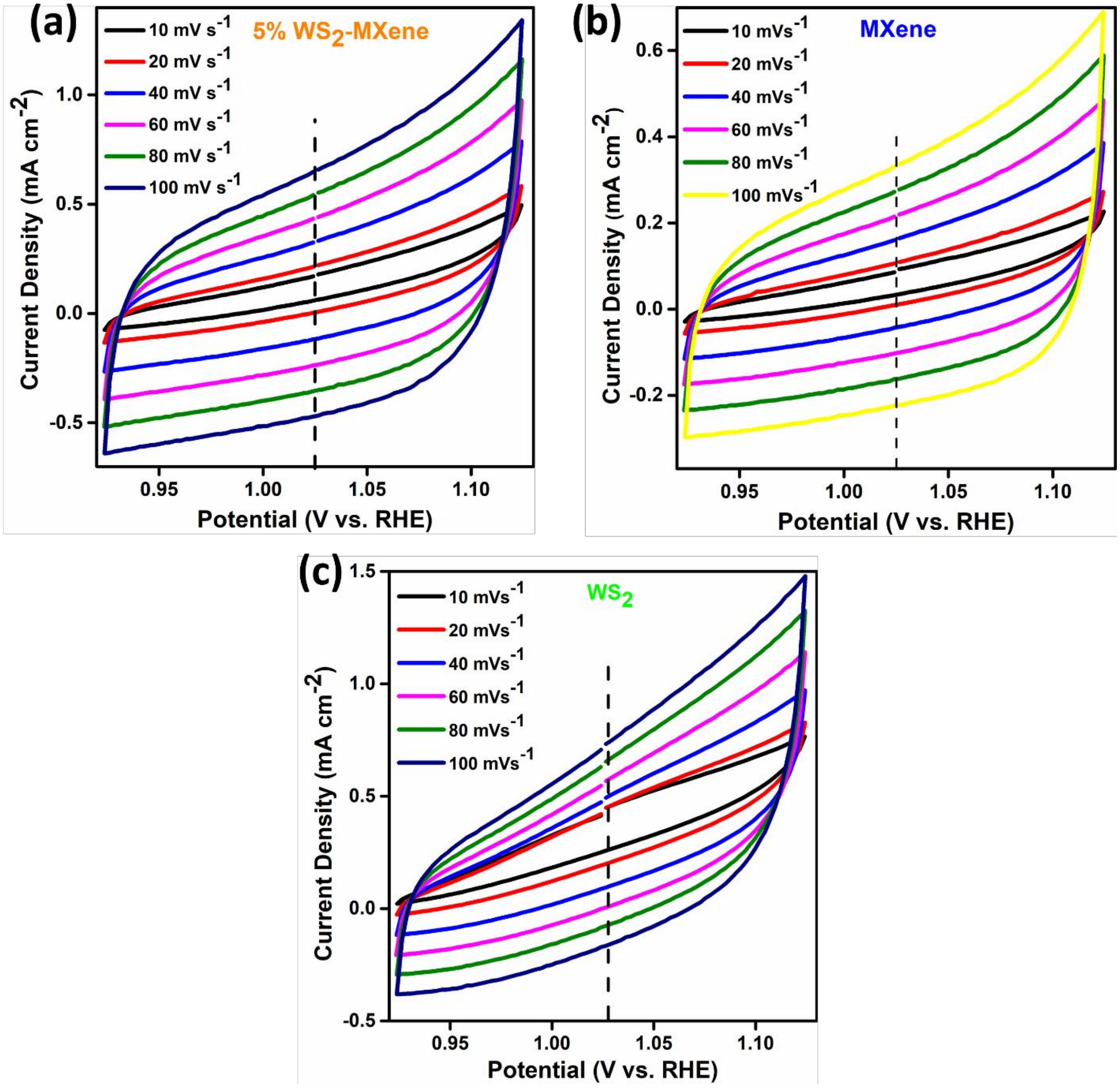

Figure S8. CV curves in 1 M KOH electrolyte for a) 5% WS_2-MXene heterostructure, b) Pristine MXene, c) Pure WS_2 at different scan rates from 10-100 mVs^{-1}.

The value of double layer capacitance (C_{dl}) was determined by plotting the change in current density (Δj) against the scan rates. Δj is the average difference between the cathodic current (j_c) and the anodic current (j_a), as shown in Eq. (S3). The slope of this plot was used to calculate the C_{dl} value.[2,3]

$$\Delta j = \frac{j_c - j_a}{2} \qquad \text{(S3)}$$

Furthermore, the CV curves were obtained by varying the scan rate from 10 to 100 mVs^{-1} for each sample. The active sites of the electrocatalysts were identified by determining the electrochemical surface area (ECSA) using Eq. S4.[4]

$$\mathrm{ECSA} = \frac{C_{dl}}{C_s} \quad \text{(S4)}$$

Where, the specific capacitance (Cs) is given by Eq. 5.

$$C_s = \frac{Q}{mV} = \frac{A}{2\,m\,k\,(V_2 - V_1)} \quad \text{(S5)}$$

Where Q is the capacitive charge was computed from the area beneath the CV sweep at 100 mVs^{-1} for the 5% WS_2-MXene, MXene, and WS_2 samples. Scan rate is equal to k (0.1 Vs^{-1}), m is the mass of the catalyst deposited (3 mg), and ΔV (V_2-V_1) is the potential window (0.2 V) for CV scan.

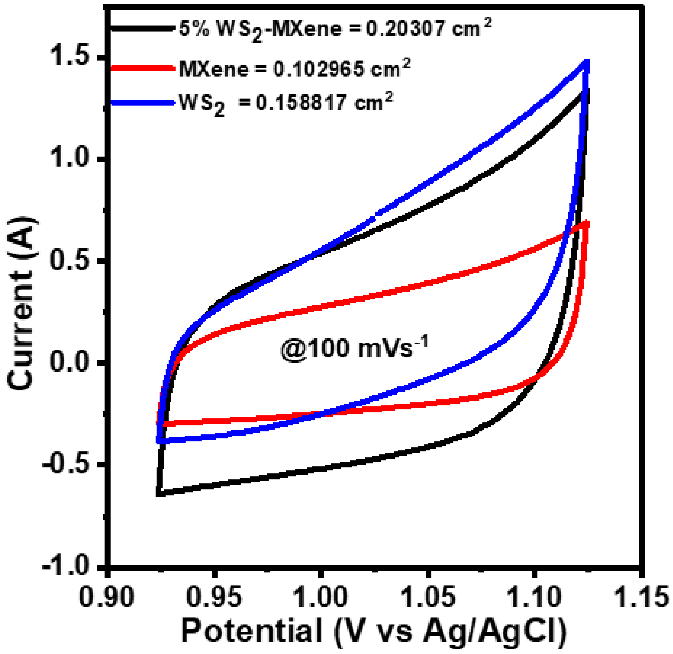

Figure S9. Cyclic Voltammetry plots in the non-faradaic region at the scan rate of 100 mVs^{-1} for pure $Ti_3C_2T_x$ MXene; WS_2; and 5% WS_2-$Ti_3C_2T_x$ MXene; exhibiting the highest area of the loop in 5% WS_2-$Ti_3C_2T_x$ MXene.

The turnover frequency (TOF) has been calculated using the Eq. S6.[5]

$$\mathrm{TOF} = \frac{i}{2\,n\,F} \quad \text{(S6)}$$

Where i is the current at -0.8 V vs RHE from LSV, F is the Faraday's constant (96500 $C.mol^{-1}$) and n is the active site density that is calculated from Eq. (S7).

$$n = \frac{Q}{2\,F} \qquad (S7)$$

$$Q = \frac{\text{Area in the CV}}{\textit{Scan Rate}} \qquad (S8)$$

Table S3. Table showing the resistance to charge transfer for the as-prepared electrocatalyst.

Electrocatalyst	R_{ct} / Ohm
5% WS_2-MXene	2.56
2% WS_2-MXene	2.58
MXene	2.60
15% WS_2-MXene	2.68
WS_2	4.07

Table S4. Comparison of HER performance of the 2D-2D heterostructure electrocatalyst in 1 M KOH.

2D-2DElectrocatalyst	Overpotential@ 10 mA cm^{-2} (mV)	Tafel slope ($mVdec^{-1}$)	Electrolyte	References
5% WS_2-MXene	60	34.9	1 M KOH	This work
$NiCo_2S_4/ReS_2$	85	78.3	1 M KOH	Pei et al.[6]
MoC–Mo_2C-790	98.2	59	1 M KOH	Liu et al.[7]
MoS_2/Ni_3S_2	110	83	1 M KOH	Zhang et al.[8]
Co_9S_8@MoS_2	190	110	1 M KOH	Zhu et al.[9]
CoS_2–MoS_2	123	86	1 M KOH	Wang et al.[10]
g-C_3N_4/rGO	183	164	1 M KOH	Choi et al.[11]
MoS_2-Nb_2CT_x	141	93.4	1 M KOH	Hu et al.[12]

$MoS_2@FePS_3$	176	127	1 M KOH	Huang et al.[13]

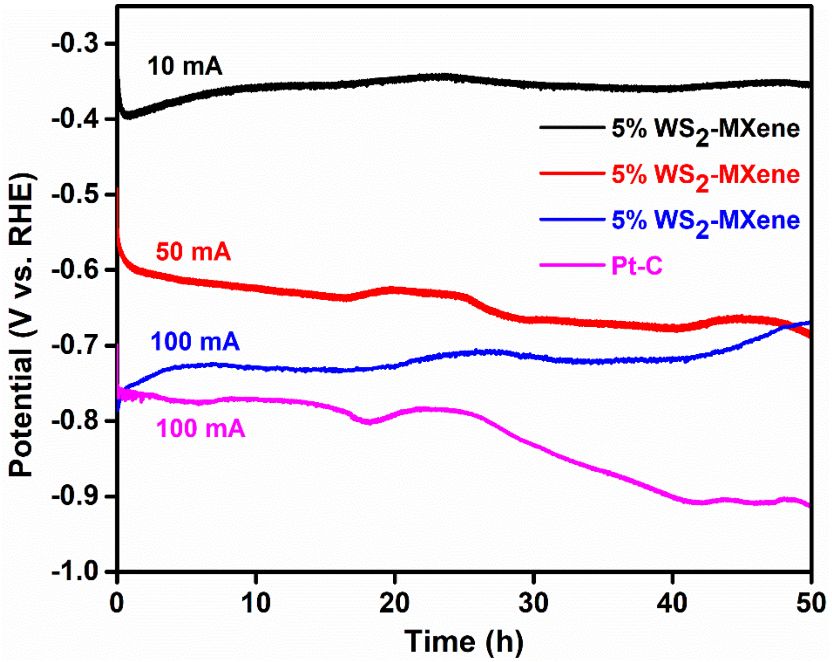

Figure S10. Durability of 5% WS_2-MXene at various current densities of 10, 50 and 100 mA cm^{-2} in comparison to the Pt/C electrocatalyst.

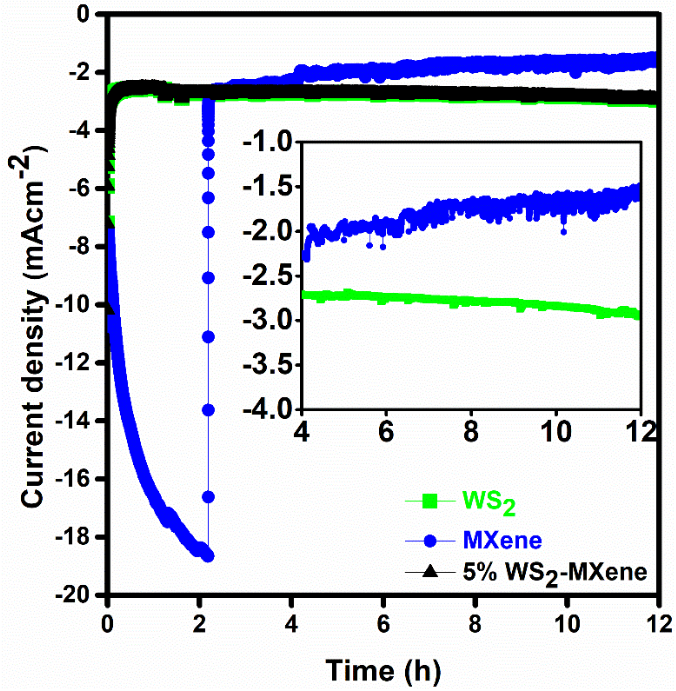

Figure S11. Chronoamperometric durability of pristine WS_2, MXene and 5% WS_2-MXene in 12 h of HER.

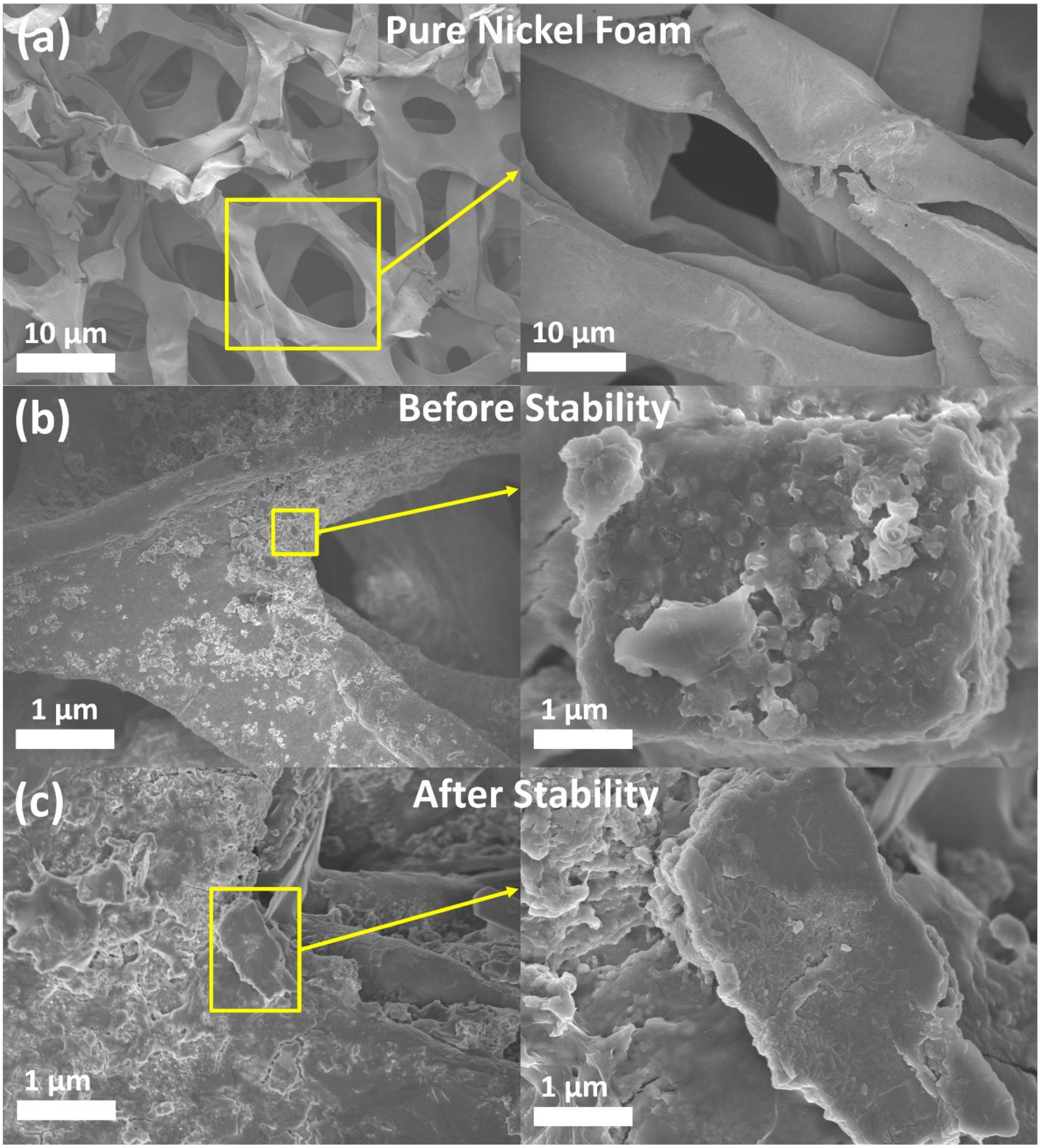

Figure S12. SEM images of (a) Pure NF, (b) 5% WS_2-MXene before stability, and (c) 5% WS_2-MXene after the durability test in 1 M KOH.

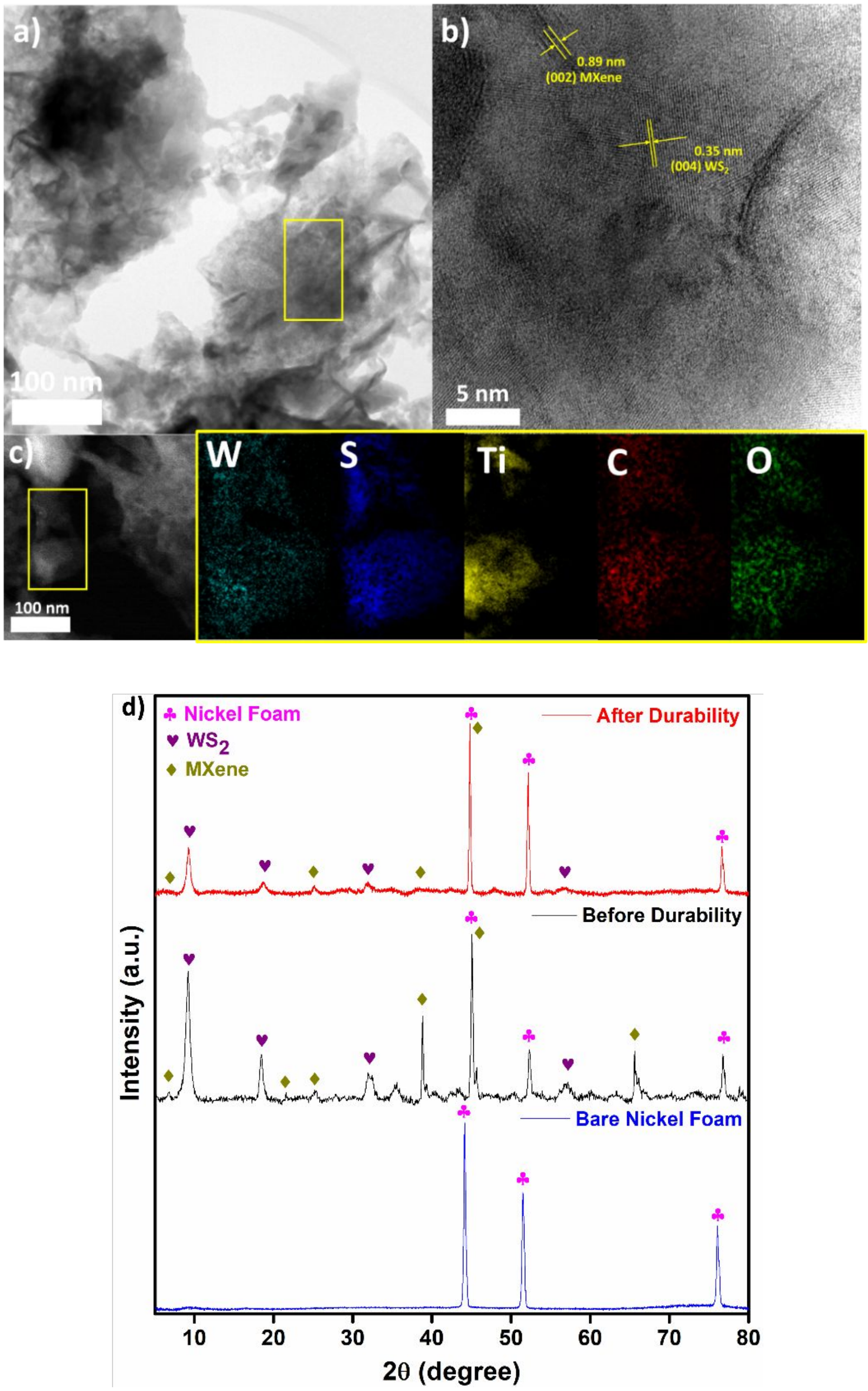

Figure S13. a) TEM image, b) highlighted region displaying HRTEM, c) EDX mapping of the highlighted region illustrating the elemental mapping of W, S, Ti, C, and O, d) XRD pattern of 5% WS_2-MXene after 50 hours of HER in an alkaline medium.

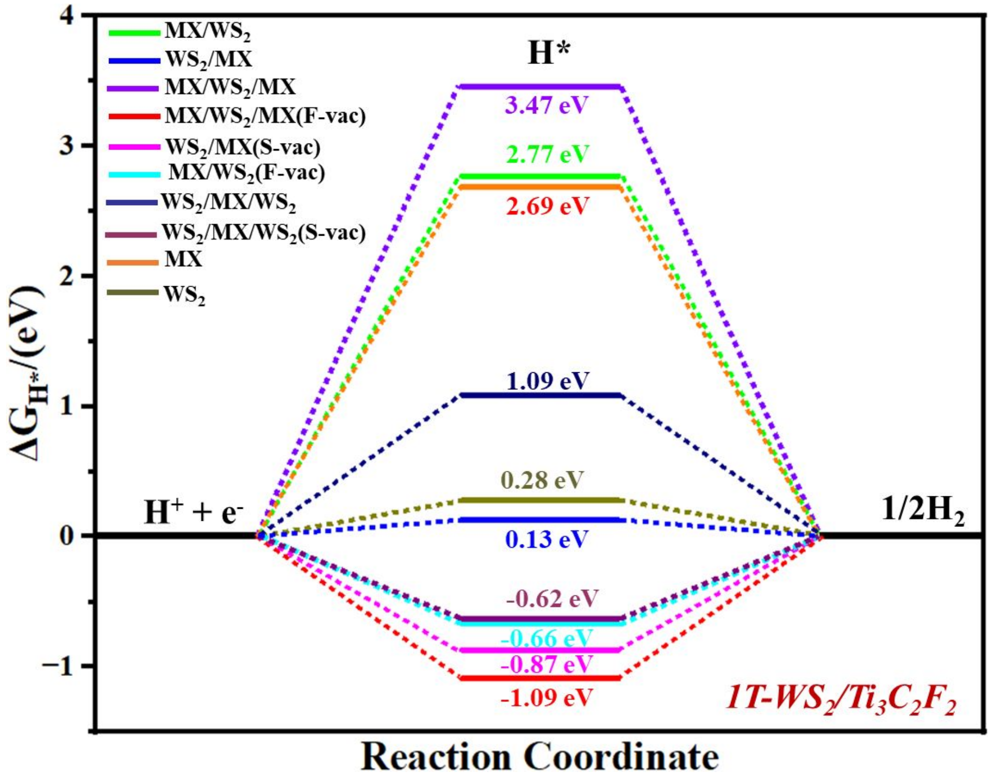

Figure S14. The corresponding Gibbs free energy profile (ΔG_{H*}) for HER at the active site on the WS_2, $Ti_3C_2T_2$, $WS_2/Ti_3C_2T_2$, $Ti_3C_2T_2/WS_2$, $WS_2/Ti_3C_2T_2$(S-vac), $Ti_3C_2T_2/WS_2$(F-vac), $Ti_3C_2T_2/WS_2/Ti_3C_2T_2$, $Ti_3C_2T_2/WS_2/Ti_3C_2T_2$(F-vac), $WS_2/Ti_3C_2T_2/WS_2$ and $WS_2/Ti_3C_2T_2/WS_2$(S-vac) surfaces. The absolute value of ΔG_{H*} for HER activity is close to zero ($\Delta G_{H*} \rightarrow 0$).

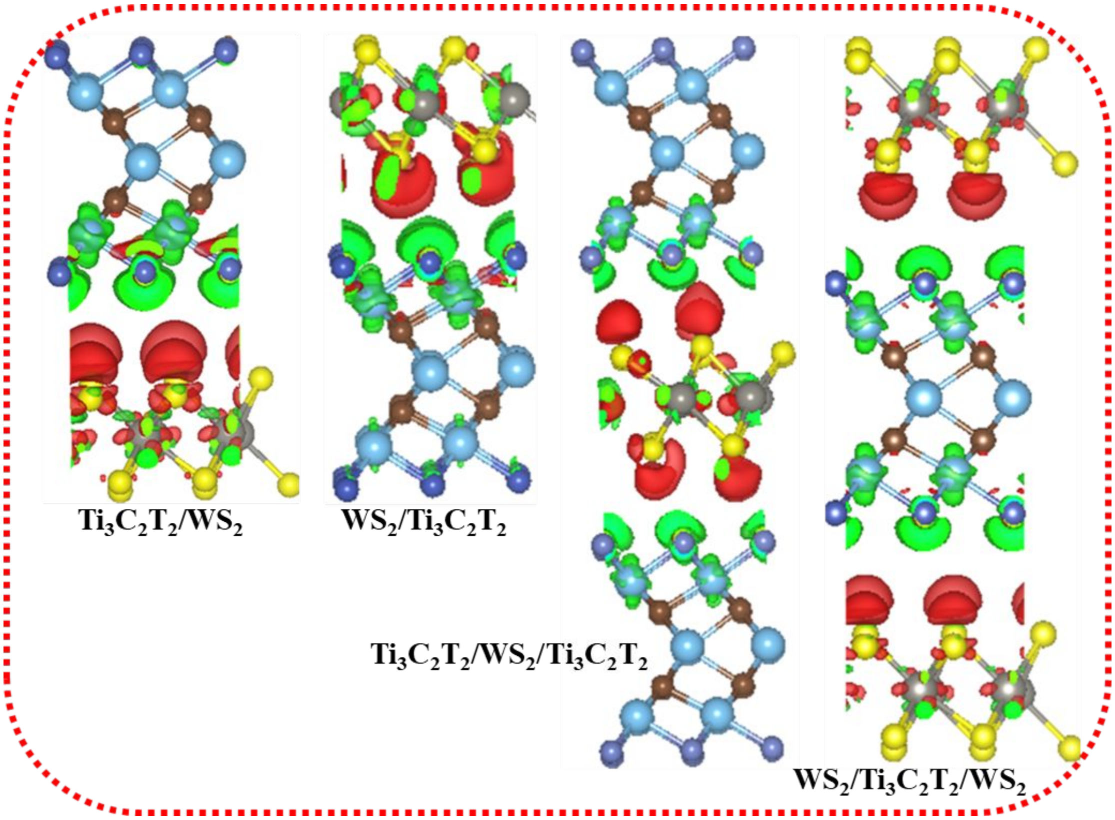

Figure S15. Charge density difference for, $Ti_3C_2T_2/WS_2$, $WS_2/Ti_3C_2T_2$, $Ti_3C_2T_2/WS_2/Ti_3C_2T_2$, and $WS_2/Ti_3C_2T_2/WS_2$ hybrid structures.

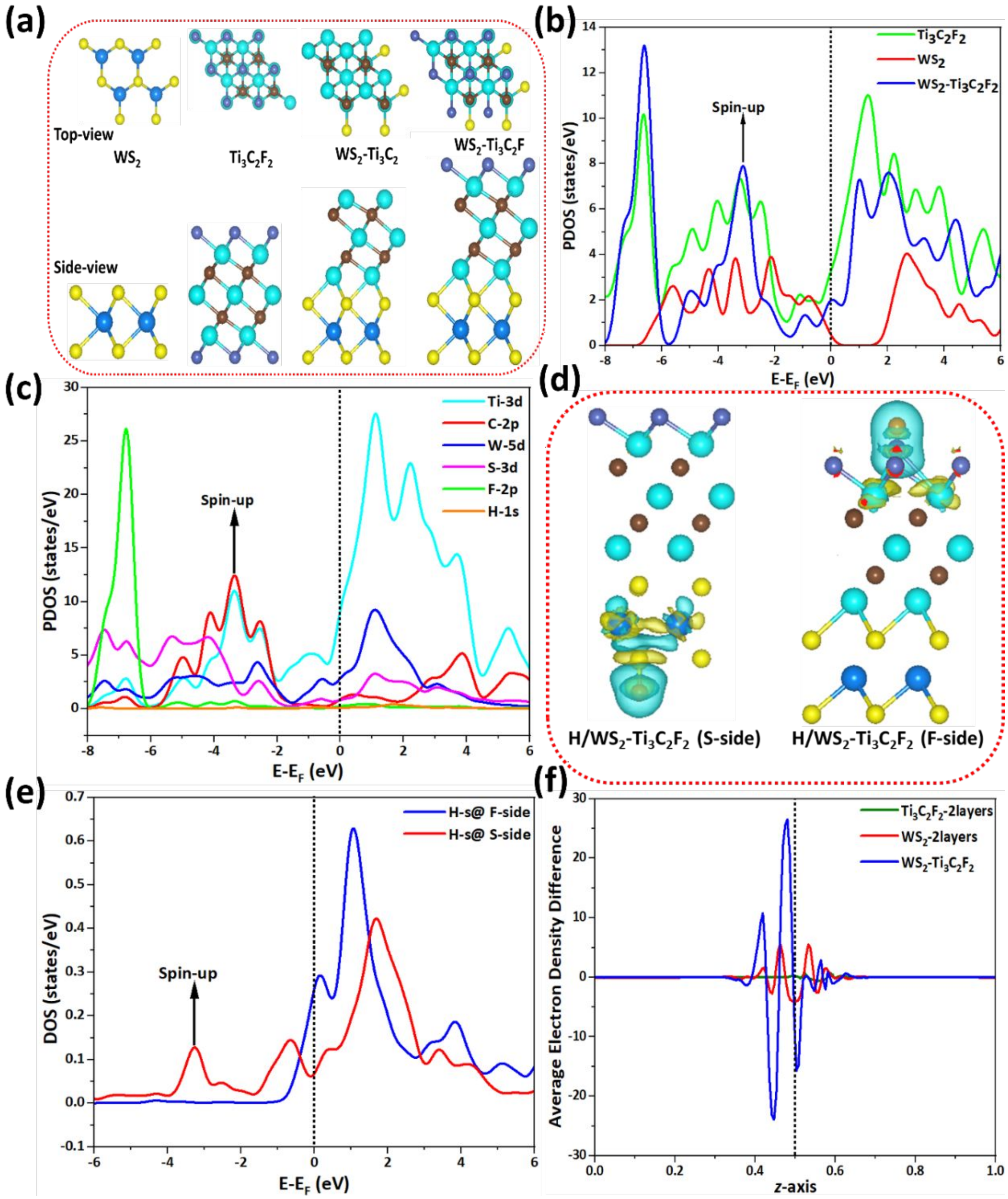

Figure S16. (a) Top and side views of optimized configurations of 2H-WS_2, $Ti_3C_2F_2$ and hybrid structure 2H-WS_2-$Ti_3C_2F_2$. (b) Total density of states for hybrid structure 2H-WS_2-$Ti_3C_2F_2$ and individual structure 2H-WS_2 and $Ti_3C_2F_2$. (c) Projected DOS of the hybrid structure with individual atoms contribution. (d) CDD for hydrogen adsorbed at F and S site, electrons depletion and accumulation are revealed in blue and yellow, respectively (e) Density of states for hydrogen adsorbed at F and S site of 2H-WS_2-$Ti_3C_2F_2$. (f) Average charge accumulation at the interface between $Ti_3C_2F_2$ and 2H-WS_2 in the hybrid structure, two layers of $Ti_3C_2F_2$ and two layers of 2H-WS_2.

The binding nature and thermodynamic stability are assessed using partial density of states (PDOS) and charge density difference (CDD). **Figure S16(b)** illustrates the total density of states of 2H-WS_2, $Ti_3C_2F_2$ and hybrid structure 2H-WS_2-$Ti_3C_2F_2$. We found that the total density of states for the hybrid structure WS_2-$Ti_3C_2F_2$ exhibits better metallic behavior in comparison to $Ti_3C_2F_2$ and WS_2, which have a broader band gap. The presence of strong peaks near to the Fermi level for both the hybrid structure and $Ti_3C_2F_2$ specifies a high chemical reactivity, which improves HER performance. We have anticipated the spin-polarized density of states projected on the several atomic contributions (Ti-3d, C-2p, F-2p, S-3d, H-1s and W-5d) to better understand the binding nature between the hybrid structure as shown in **Figure S16c**. According to the PDOS, the major peaks of Ti-3d orbital close to the Fermi level (E_F) is evidence of the high reactivity which might be responsible for the activation of adsorbates during catalysis reactions.[83,84]

Close to the E_F, the extreme closeness of W-5d orbital with the S-3p is because of their relatively robust p-d orbital hybridization, that consequently influence the electron transfer from the WS_2 to $Ti_3C_2F_2$.[85] Moreover, the CDD of the adsorbed H* atom on the hybrid structure and between the two layers of WS_2, $Ti_3C_2F_2$ and WS_2-$Ti_3C_2F_2$ **(see Figure S17)** were also measured to get insight into the nature of chemical bonding between the adsorbate and interatomic layers, the results are presented in **Figure S16 (d) and S18**. As seen from **Figure S16(d)**, the adsorption of hydrogen on S and F sites affects the distribution of significant charge on the beneath atomic layers (W and Ti). We found that the S site's hydrogen adsorption influences the charge distribution of both the S and W atoms, but the F site only distributes the F layer with no impact on the Ti layer. As previously indicated by PDOS, this is mainly attributed to strong p-d orbital hybridization between W and S. The HER activities on both sites (S and F site) are further confirm the preceding arguments by calculating the PDOS **(Figure S16(e))**. Spin-polarized partial density of states results show that the adsorbed H-1s orbital above the E_F is lower on the S-site compared to the F-site, indicating a better interaction with the H* and improves the HER performance. Likewise, the average electron density difference plot **(Figure S16(f))** further confirmed that the hybrid structure WS_2-$Ti_3C_2F_2$ have more charge distribution than $Ti_3C_2F_2$ and WS_2, and has better catalytic activity for hybrid structures.

Since the electron transfer has a significant impact on HER performance, the WS_2 and $Ti_3C_2F_2$ work functions were calculated using DFT. In order to calculate the Fermi level (E_F) and work function (Φ), the average potential profiles along the z-axis of $Ti_3C_2F_2$ and WS_2 were considered **(Figure S19)**. The calculated Fermi-level and work function of $Ti_3C_2F_2$ ($E_F = -0.194$ eV,$\Phi = 4.135$ eV) and 2H-WS_2 ($E_F = -3.58$ eV,$\Phi = 5.478\ eV$), respectively. When these two materials are in contact, charge transfer from WS_2 to $Ti_3C_2F_2$ according to the E_F and Φ difference. The Schottky junction between $Ti_3C_2F_2$ and WS_2 creates an electric field when they are in contact **(Figure S19a-b)**. Electric fields at interfaces increase charge separation, transport, and promote better HER activities.[86] This is manifested by the WS_2-$Ti_3C_2F_2$

composite's reduced resistance. Additionally, the charge accumulation across the interface of WS_2-$Ti_3C_2F_2$ is greater than that across the stack of $Ti_3C_3F_2$ and WS_2 individually **(Figure S18)**.

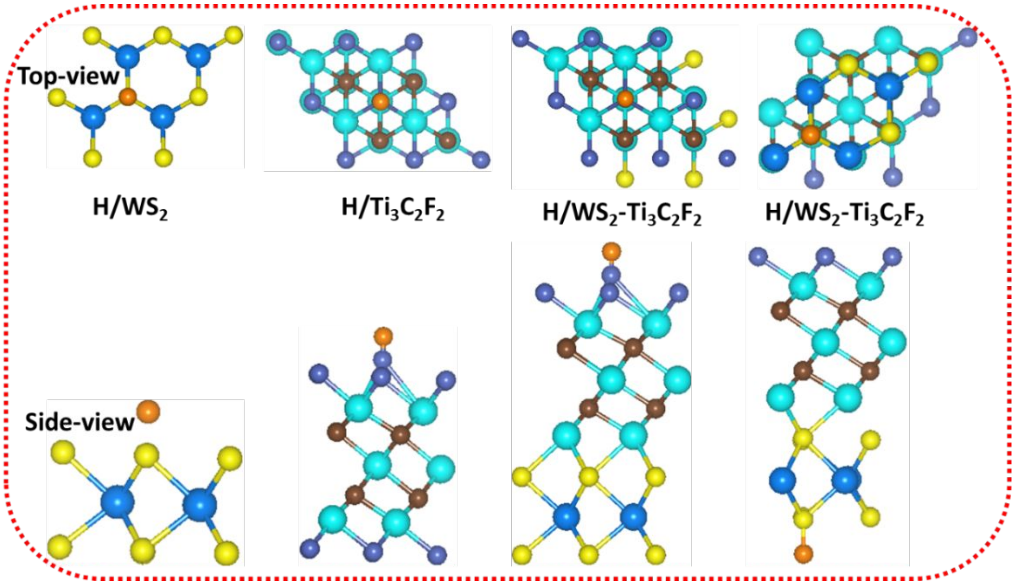

Figure S17. Top and side views of optimized configurations of hydrogen adsorbed on the 2H-WS_2, $Ti_3C_2F_2$, hybrid structure 2H-WS_2-$Ti_3C_2F_2$ (F-side) and 2H-WS_2-$Ti_3C_2F_2$ (S-side).

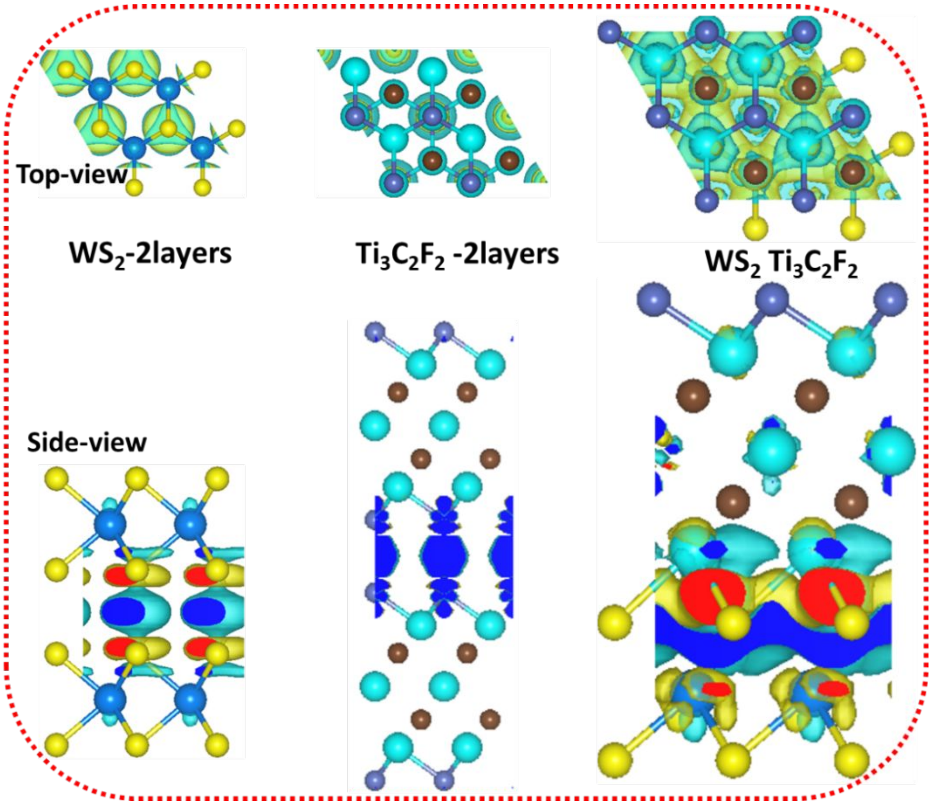

Figure S18. Charge density difference for, two layers of 2H-WS_2, two layers of $Ti_3C_2F_2$ and 2H-WS_2-$Ti_3C_2F_2$ hybrid structure.

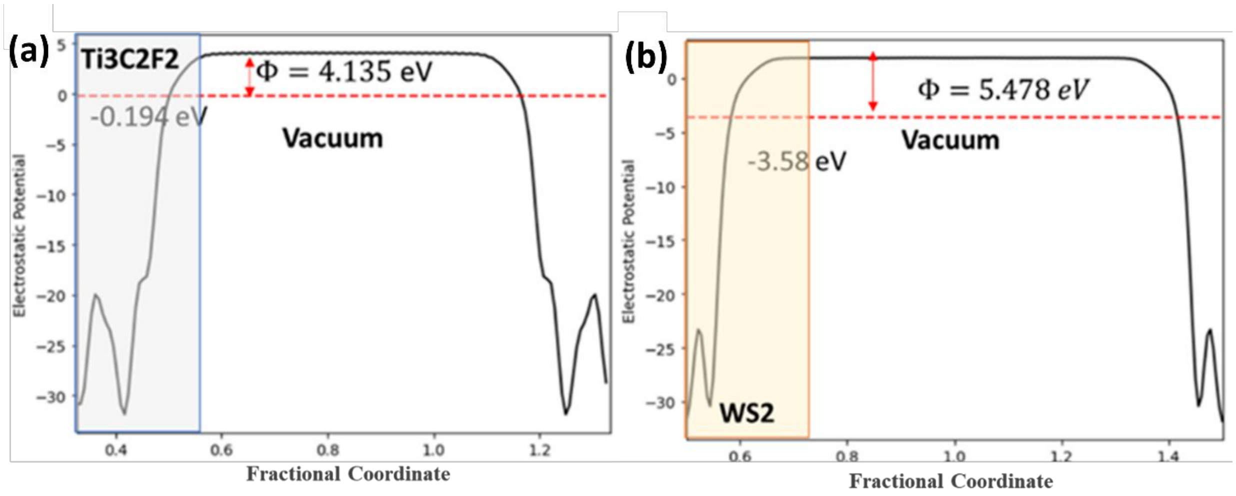

Figure S19. Electrostatic potential (a) $Ti_3C_2F_2$ and (b) 2H-WS_2.

References

1) Bandara, N.; Esparza, Y.; Wu, J. Graphite Oxide Improves Adhesion and Water Resistance of Canola Protein–Graphite Oxide Hybrid Adhesive. *Sci. Rep.* **2017**, *7* (1), 11538. https://doi.org/10.1038/s41598-017-11966-8.
2) Huang, L.; Hou, Y.; Yu, Z.; Peng, Z.; Wang, L.; Huang, J.; Zhang, B.; Qian, L.; Wu, L.; Li, Z. Pt/Fe-NF Electrode with High Double-Layer Capacitance for Efficient Hydrogen Evolution Reaction in Alkaline Media. *Int. J. Hydrog. Energy* **2017**, *42* (15), 9458–9466. https://doi.org/10.1016/j.ijhydene.2017.02.055.
3) Yan, P.; Wu, Y.; Wei, X.; Zhu, X.; Su, W. Preparation of Robust Hydrogen Evolution Reaction Electrocatalyst WC/C by Molten Salt. *Nanomaterials* **2020**, *10* (9), 1621. https://doi.org/10.3390/nano10091621.
4) Xing, J.; Zou, Z.; Guo, K.; Xu, C. The Effect of Phosphating Time on the Electrocatalytic Activity of Nickel Phosphide Nanorod Arrays Grown on Ni Foam. *J. Mater. Res.* **2018**, *33* (5), 556–567. https://doi.org/10.1557/jmr.2017.399.
5) Riyajuddin, S.; Azmi, K.; Pahuja, M.; Kumar, S.; Maruyama, T.; Bera, C.; Ghosh, K. Super-Hydrophilic Hierarchical Ni-Foam-Graphene-Carbon Nanotubes-Ni2P–CuP2 Nano-Architecture as Efficient Electrocatalyst for Overall Water Splitting. *ACS Nano* **2021**, *15* (3), 5586–5599. https://doi.org/10.1021/acsnano.1c00647.
6) Pei, C.; Kim, M.; Li, Y.; Xia, C.; Kim, J.; So, W.; Yu, X.; Park, H. S.; Kim, J. K. Electron Transfer-Induced Metal Spin-Crossover at $NiCo_2S_4/ReS_2$ 2D–2D Interfaces for Promoting PH-universal Hydrogen Evolution Reaction. *Adv. Funct. Mater.* **2023**, *33* (4), 2210072. https://doi.org/10.1002/adfm.202210072.
7) Liu, W.; Wang, X.; Wang, F.; Du, K.; Zhang, Z.; Guo, Y.; Yin, H.; Wang, D. A Durable and PH-Universal Self-Standing MoC–Mo2C Heterojunction Electrode for Efficient Hydrogen Evolution Reaction. *Nat. Commun.* **2021**, *12* (1), 6776. https://doi.org/10.1038/s41467-021-27118-6.
8) Zhang, J.; Wang, T.; Pohl, D.; Rellinghaus, B.; Dong, R.; Liu, S.; Zhuang, X.; Feng, X. Interface Engineering of MoS_2/Ni_3S_2 Heterostructures for Highly Enhanced Electrochemical Overall-Water-Splitting Activity. *Angew. Chem. Int. Ed.* **2016**, *55* (23), 6702–6707. https://doi.org/10.1002/anie.201602237.

9) Zhu, H.; Zhang, J.; Yanzhang, R.; Du, M.; Wang, Q.; Gao, G.; Wu, J.; Wu, G.; Zhang, M.; Liu, B.; Yao, J.; Zhang, X. When Cubic Cobalt Sulfide Meets Layered Molybdenum Disulfide: A Core-Shell System Toward Synergetic Electrocatalytic Water Splitting. *Adv. Mater.* **2015**, *27* (32), 4752–4759. https://doi.org/10.1002/adma.201501969.
10) Wang, Y.; Zhu, Y.; Afshar, S.; Woo, M. W.; Tang, J.; Williams, T.; Kong, B.; Zhao, D.; Wang, H.; Selomulya, C. One-Dimensional CoS2–MoS2 Nano-Flakes Decorated MoO2 Sub-Micro-Wires for Synergistically Enhanced Hydrogen Evolution. *Nanoscale* **2019**, *11* (8), 3500–3505. https://doi.org/10.1039/C8NR08418A.
11) (Choi, H.; Surendran, S.; Sim, Y.; Je, M.; Janani, G.; Choi, H.; Kim, J. K.; Sim, U. Enhanced Electrocatalytic Full Water-Splitting Reaction by Interfacial Electric Field in 2D/2D Heterojunction. *Chem. Eng. J.* **2022**, *450*, 137789. https://doi.org/10.1016/j.cej.2022.137789.
12) Hu, L.; Sun, Y.; Gong, S.-J.; Zong, H.; Yu, K.; Zhu, Z. Experimental and Theoretical Investigation on MoS_2 /MXene Heterostructure as an Efficient Electrocatalyst for Hydrogen Evolution in Both Acidic and Alkaline Media. *New J. Chem.* **2020**, *44* (19), 7902–7911. https://doi.org/10.1039/D0NJ00956C.
13) Huang, H.; Song, J.; Yu, D.; Hao, Y.; Wang, Y.; Peng, S. Few-Layer FePS3 Decorated with Thin MoS2 Nanosheets for Efficient Hydrogen Evolution Reaction in Alkaline and Acidic Media. *Appl. Surf. Sci.* **2020**, *525*, 146623. https://doi.org/10.1016/j.apsusc.2020.146623.
14) Jin, Di, et al. "Computational screening of 2D ordered double transition-metal carbides (MXenes) as electrocatalysts for hydrogen evolution reaction." *The Journal of Physical Chemistry C* 124.19 (2020): 10584-10592.
15) Han, Ali, et al. "One-step synthesis of single-site vanadium substitution in 1T-WS2 monolayers for enhanced hydrogen evolution catalysis." *Nature Communications* 12.1 (2021): 709.
16) Kumar, Ashwani, et al. "Moving beyond bimetallic-alloy to single-atom dimer atomic-interface for all-pH hydrogen evolution." *Nature Communications* 12.1 (2021): 6766.
17) Pettersson, Lars Gunnar Moody, and Anders Nilsson. "A molecular perspective on the d-band model: Synergy between experiment and theory." *Topics in catalysis* 57 (2014): 2-13.

18) Liu, Xiaofei, et al. "Distinctive pd orbital hybridization in RuSb nanobranches for simultaneously enhanced hydrogen evolution and hydrazine oxidation in alkaline seawater." *Applied Catalysis B: Environmental* 333 (2023): 122771.
19) Zuo, Gancheng, et al. "Enhanced photocatalytic water oxidation by hierarchical 2D-Bi2MoO6@ 2D-MXene Schottky junction nanohybrid." *Chemical Engineering Journal* 403 (2021): 126328.
20) K. Ren, M. Sun, Y. Luo, S. Wang, J. Yu and W. Tang, *Applied Surface Science*, 2019, **476**, 70-75.
